# STAU2 protein level is controlled by caspases and the CHK1 pathway and regulates cell cycle progression in the non-transformed hTERT-RPE1 cells

**DOI:** 10.1186/s12860-021-00352-y

**Published:** 2021-03-04

**Authors:** Lionel Condé, Yulemi Gonzalez Quesada, Florence Bonnet-Magnaval, Rémy Beaujois, Luc DesGroseillers

**Affiliations:** Département de Biochimie et Médecine Moléculaire, Faculté de médecine, Université de Montréal, 2900 Édouard Montpetit, Montréal, QC H3T 1J4 Canada

**Keywords:** Staufen2, CHK1, Caspase, Cell proliferation

## Abstract

**Background:**

Staufen2 (STAU2) is an RNA binding protein involved in the posttranscriptional regulation of gene expression. In neurons, STAU2 is required to maintain the balance between differentiation and proliferation of neural stem cells through asymmetric cell division. However, the importance of controlling STAU2 expression for cell cycle progression is not clear in non-neuronal dividing cells. We recently showed that STAU2 transcription is inhibited in response to DNA-damage due to E2F1 displacement from the *STAU2* gene promoter. We now study the regulation of STAU2 steady-state levels in unstressed cells and its consequence for cell proliferation.

**Results:**

CRISPR/Cas9-mediated and RNAi-dependent STAU2 depletion in the non-transformed hTERT-RPE1 cells both facilitate cell proliferation suggesting that STAU2 expression influences pathway(s) linked to cell cycle controls. Such effects are not observed in the CRISPR STAU2-KO cancer HCT116 cells nor in the STAU2-RNAi-depleted HeLa cells. Interestingly, a physiological decrease in the steady-state level of STAU2 is controlled by caspases. This effect of peptidases is counterbalanced by the activity of the CHK1 pathway suggesting that STAU2 partial degradation/stabilization fines tune cell cycle progression in unstressed cells. A large-scale proteomic analysis using STAU2/biotinylase fusion protein identifies known STAU2 interactors involved in RNA translation, localization, splicing, or decay confirming the role of STAU2 in the posttranscriptional regulation of gene expression. In addition, several proteins found in the nucleolus, including proteins of the ribosome biogenesis pathway and of the DNA damage response, are found in close proximity to STAU2. Strikingly, many of these proteins are linked to the kinase CHK1 pathway, reinforcing the link between STAU2 functions and the CHK1 pathway. Indeed, inhibition of the CHK1 pathway for 4 h dissociates STAU2 from proteins involved in translation and RNA metabolism.

**Conclusions:**

These results indicate that STAU2 is involved in pathway(s) that control(s) cell proliferation, likely via mechanisms of posttranscriptional regulation, ribonucleoprotein complex assembly, genome integrity and/or checkpoint controls. The mechanism by which STAU2 regulates cell growth likely involves caspases and the kinase CHK1 pathway.

**Supplementary Information:**

The online version contains supplementary material available at 10.1186/s12860-021-00352-y.

## Background

RNA-binding proteins (RBPs) and the posttranscriptional regulation of gene expression that they impose on their associated RNAs play crucial role in cells [1–3]. They group mRNAs within regulons coding for functionally-related proteins and allow a timely control of protein synthesis in response to changing cellular environments [4]. RBPs are involved in diverse biological processes that include cell proliferation, DNA damage response and metabolism [5]. Given the broad spectrum of biological functions that depend on RBP activity, it is not surprising that their overexpression or depletion result in strong cellular phenotypes, indicating that their expression levels have to be tightly controlled. Moreover, mutations in genes that code for RBPs are linked to numerous diseases including neurological disorders and various types of cancer [6, 7]. However, these RBPs are part of intricate pathways in which several RBPs and RNAs associated to form functional RNA regulons involved in biological functions. Understanding the role of each molecule in these complexes and how their mutation affects specific biological systems to cause diseases is crucial to design eventual appropriate therapy.

Staufen2 (STAU2) is an RNA-binding protein [8, 9] that binds mRNAs coding for proteins involved in multiple cellular processes including cell cycle regulation [10]. The *STAU2* gene, through differential splicing, generates several isoforms, the major ones having molecular masses of 52, 59 and 62 kDa [9]. STAU2 isoforms are mostly cytoplasmic, localizing near the endoplasmic reticulum [9], but can also be found in the nucleus and nucleolus [11]. STAU2 regulates mRNA expression through several posttranscriptional molecular processes such as mRNA localization, differential splicing, regulation of translation, and mRNA decay [12–16]. The physiological consequences of STAU2 downregulation was studied in several animal models. In zebrafish, Stau2 is required for the migration of primordial germ cells and for survival of neurons in the central nervous system [17]. In Xenopus, Stau2 controls the anterior endodermal organ formation [18]. In mouse oocytes, STAU2 is needed for meiosis progression and spindle integrity [19]. In chicken, STAU2 downregulation induces small eye development as a consequence of reduced cell proliferation [20]. Likewise, in developing mouse cortex, STAU2 regulates the balance between neural stem cell maintenance and differentiation [21, 22]: STAU2 downregulation induces cell differentiation while its overexpression produces periventricular neuronal masses. STAU2 depletion in mouse and rat brains impairs hippocampal spatial working memory, spatial novelty detection and/or associative learning and memory [23, 24]. This is consistent with the importance of STAU2 for dendritic spine morphogenesis and for long-term synaptic depression [13, 15]. STAU2 is also linked to the DNA damage response and the apoptotic pathway since STAU2 depletion causes an accumulation of DNA damage and facilitates apoptosis in the HCT116 cancer cell line [25]. In addition, induction of single-stranded break causes the inhibition of STAU2 transcription as a consequence of delocalization of the transcription factor E2F1 from the *STAU2* promoter [25]. However, its biological importance and its regulation in unstressed cells are still uncharacterized.

In this paper, we now show that STAU2 knockout facilitates cell proliferation in the non-transformed hTERT-RPE1 cells. We further show that STAU2 protein steady-state level is stabilized by the CHK1 pathway and is decreased by the activity of caspases in unstressed cells. Finally, a genome wide approach reveals that STAU2 is in close proximity to proteins involved in the posttranscriptional regulation of gene expression and to proteins of the nucleolus, including proteins linked to ribosome biogenesis and to DNA repair. Altogether, our results identify novel STAU2 functions at the crossroad of cell cycle regulation and DNA damage response.

## Results

### STAU2 depletion facilitates cell growth of non-transformed hTERT-RPE1 cells

To study the role of STAU2 in unstressed cells, we first generated STAU2 knockout (STAU2-KO) cells using the non-transformed hTERT-RPE1 cells and the CRISPR/Cas9 technology (Supp Fig. S1). Cells were transfected with a plasmid expressing CRISPR/Cas9 and an RNA guide targeting exon 6 of the *STAU2* gene. This exon is just downstream of the AUG initiation codon and common to all STAU2 isoforms. Individual clones were isolated and tested for STAU2 expression by dot blotting (Supp Fig. S1B) revealing that 26% of the selected clones were negative for STAU2 expression. DNA sequencing of *STAU2* exon 6 of two STAU2-KO clones showed the presence of short deletions that introduced premature stop codons (Supp Fig. S1A). RT-qPCR quantification revealed that STAU2 mRNA levels were also decreased in all tested clones compared to that in wild type (WT) cells (Supp Fig. S1C).

**Fig. 1:**
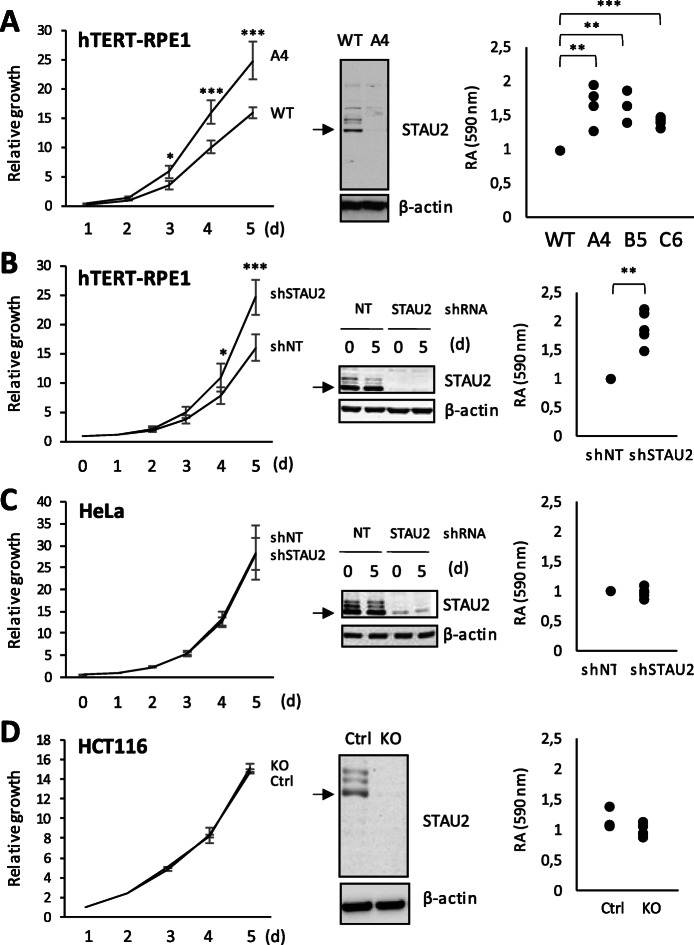
STAU2 depletion facilitates cell growth of hTERT-RPE1 cells. Growth curves (left panels) and colony growth assays (right panels) were used to monitor cell proliferation. The relative growth of wild-type cells was arbitrary fixed to 1 for the colony assays. *** *p*-value ≤0.001; ** *p*-value ≤0.01; * *p*-value ≤0.05. Two-tailed Student’s *t*-test for growth curves analysis. One sample *t*-test for the colony assay. (Middle) Representative Western blots showing STAU2 expression. **a** STAU2-KO clones (A4, B5, C6) and wild-type (WT) hTERT-RPE1 cells. *n* = 4. **b** hTERT-RPE1 cells were infected with viruses expressing shRNA control (shNT) or shRNA against STAU2 (shSTAU2). *n* = 5. **c** HeLa cells were infected with viruses expressing shRNA control (shNT) or shRNA against STAU2 (shSTAU2). *n* = 5. **d** CRISPR-mediated STAU2-KO HCT116 (STAU2-KO) and CRISPR-treated cells that still express STAU2 as controls (Ctrl). *n* = 5

To determine the impact of STAU2 knockout on cell proliferation, we studied the growth of several STAU2-KO clones compared to that of WT hTERT-RPE1 cells (Fig. 1a). Using the growth curve assay, we first showed that the STAU2-KO clone A4 proliferated faster than wild type cells, indicating that STAU2 depletion interferes with cell cycle progression and potentiates cell growth (Fig. 1a). To rule out a putative off-target effect, we used the colony growth assay to monitor proliferation of additional STAU2-KO clones. Cell growth was significantly increased in the three tested clones (Fig. 1a) compared to WT hTERT-RPE1 cells. A similar increase in cell growth was observed in shRNA-mediated STAU2 depleted cells (Fig. 1b; Supp Fig. S2). Interestingly, STAU2 depletion in HeLa tumor cells and STAU2-KO in the HCT116 cancer cell line (Supp Fig. S3) had no effect on cell proliferation (Fig. 1c,d. Supp Fig. S2) indicating that cancer cells can bypass the consequences of STAU2 depletion that are observed in non-transformed cells.

### Caspases are involved in STAU2 degradation

**Fig. 2:**
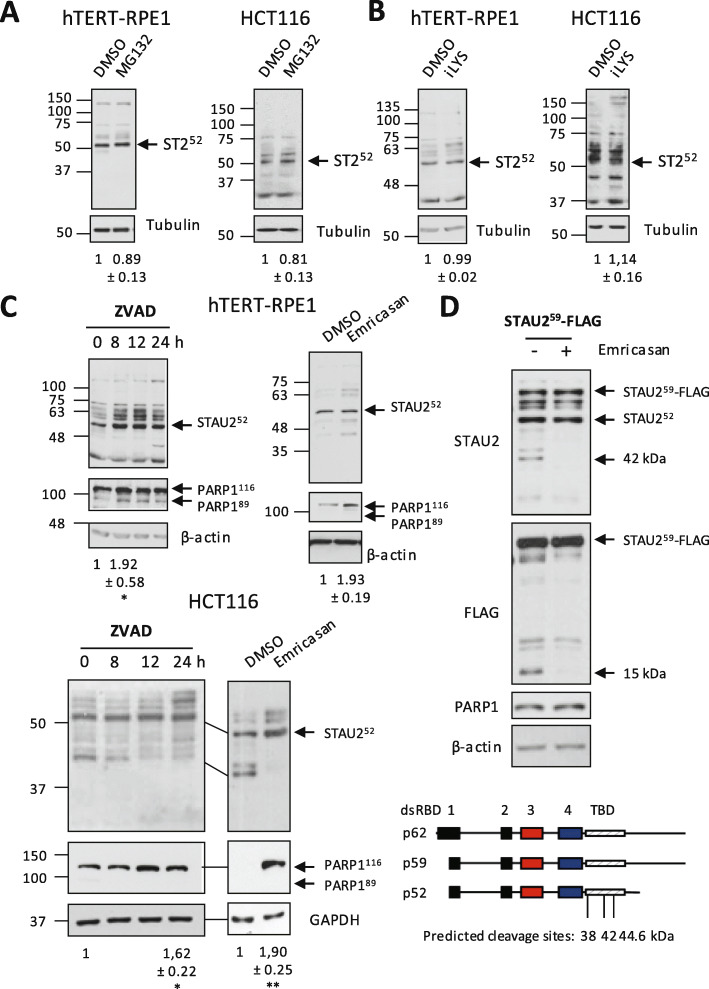
STAU2 is a target of caspase(s). Cells were incubated in the presence of different peptidase inhibitors as indicated or DMSO (vehicle). Cell extracts were analyzed by Western blotting for STAU2 expression. All Western blots are representative of at least three independently performed experiments that gave similar results. Quantification of STAU2 protein levels is indicated below the gels. The ratio of STAU2 on the loading control in DMSO-treated cells was fixed to 1. * *p*-value ≤0.05; ** *p*-value ≤0.01. One sample *t*-test. PARP1 was used as a measure of caspase inhibition. **a**, **b** hTERT-RPE1 and HCT116 cells were incubated in the presence of the proteasome inhibitor MG132 (10 μM) for 6 h (**a**) or the lysosome inhibitor iLYS (10 μM) for 10 h (**b**). **c** hTERT-RPE1 (top) and HCT116 (bottom) cells were incubated in the presence of caspase inhibitors: ZVAD-FMK (50 μM for 0, 8, 12, 24 h) or emricasan (40 μM for 24 h). **d** HCT116 cells were transfected with a plasmid coding for STAU2^59^-FLAG_3_. Cells were then incubated in the presence of DMSO (−) or of the caspase inhibitor Emricasan (40 μM) (+) for 24 h. Full length and degradation fragments were visualized by anti-STAU2 and anti-FLAG antibodies to detect the N- and C-terminal ends of STAU2, respectively. Western blots are representative of two independently performed experiments. β-actin was used as a loading control. (bottom) Schematic representation of STAU2 protein. Predicted caspase cleavage sites are shown. Red and blue boxes: major and minor RNA-binding domains, respectively. Black boxes, regions with RNA-binding consensus sequence but lacking RNA-binding activity in vitro. Hatched boxes, tubulin-binding domain

These results suggest that physiological modulation of the steady-state levels of STAU2 may be advantageous for the fine tuning of cell proliferation in changing cell environment. Therefore, to identify endogenous pathway(s) that control(s) the physiological stability/degradation of STAU2, we first tested pharmacological inhibitors known to target the major protein degradation pathways. hTERT-RPE1 and HCT116 cells were incubated with the proteasome inhibitor MG132 (Fig. 2a) for 6 h or the lysosome inhibitor iLYS (Fig. 2b) for 10 h. Western blotting experiments showed that neither the proteasome inhibitor nor the lysosome inhibitor changed the steady-state levels of STAU2.

Western blots of HCT116 cells using anti-STAU2 antibody often revealed the presence of a specific band of around 42 kDa (Fig. 2a) that was not observed in STAU2-KO cells (Fig. 1d), suggesting a partial cleavage of STAU2 by specific peptidases in unstressed cells. Therefore, as STAU2 functions are linked to cell proliferation and apoptosis [25], we asked whether caspases, whose activities are, among others, linked to these processes [26], can modulate the steady-state levels of STAU2. Therefore, hTERT-RPE1 and HCT116 cells were treated with the pan-caspase inhibitor ZVAD-FMK for increasing periods of time (Fig. 2c). Western blot analysis indicated that the steady-state levels of STAU2 increased, indicating that caspases contribute to STAU2 degradation in unstressed cells (Fig. 2c). In hTERT-RPE1 cells, STAU2 stabilization can be observed after 8 h of treatment. In HCT116 cells, although STAU2 stabilization was observed at 24 h, the disappearance of the 42 kDa fragment began at 8 h of ZVAD treatment. Incubation of these cells with another caspase inhibitor (emricasan) also protected STAU2 from degradation (Fig. 2c). Inhibition of caspases was confirmed by the stabilization of PARP1, a known target of caspase 3. Concomitant with the stabilization of the full-length STAU2 protein, the 42 kDa band recognized with anti-STAU2 antibody in untreated HCT116 cells totally disappeared upon caspases inhibition. As the anti-STAU2 antibody recognized degradation products containing the N-terminal end of STAU2, we also expressed STAU2^59^-FLAG_3_ in HCT116 cells and used the anti-FLAG antibody in an attempt to detect C-terminal fragments. In the presence of pan-caspase inhibitor, specific degradation products of around 42 and 15 kDa disappeared when using anti-STAU2 and anti-FLAG antibodies, respectively (Fig. 2d). These results identify at least one putative caspase-cleavage site in the C-terminal end of STAU2. Computer prediction identified three caspase sites that are compatible with the size of the degradation products (Fig. 2d). Altogether, these results indicate that STAU2 is a substrate of caspases in unstressed cells.

### STAU2 is in close proximity to proteins involved in RNA post-transcriptional regulation

**Fig. 3:**
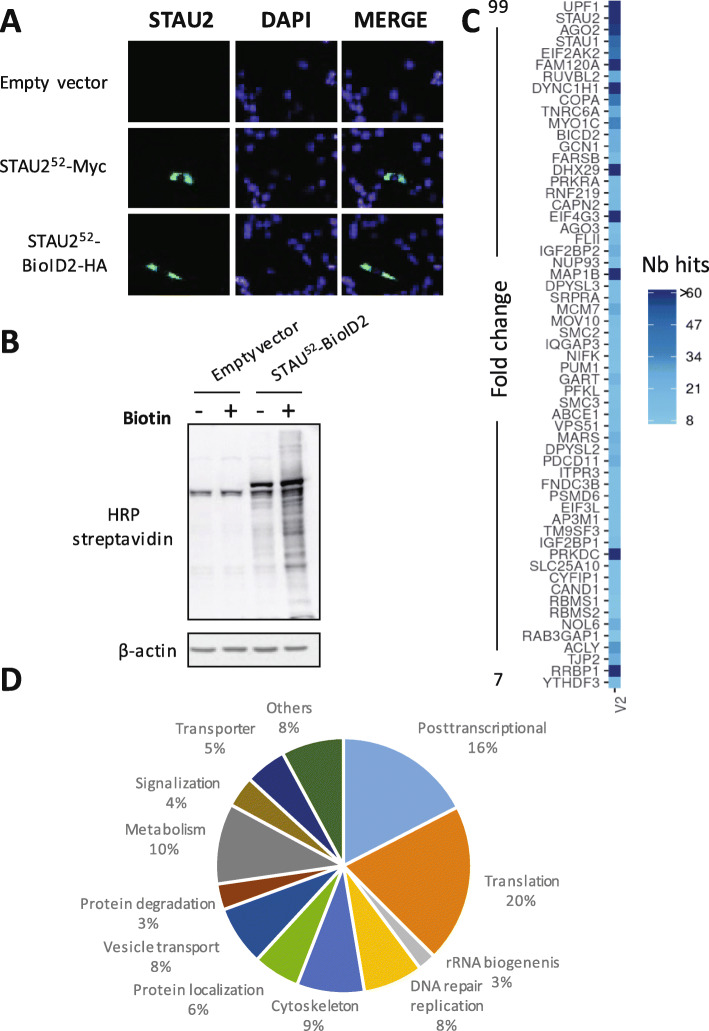
Identification of proteins in proximity to STAU2. **a** Control (empty vector), STAU2^52^-myc_3_ and STAU2^52^-BioID2-HA infected hTERT-RPE1 cells were analyzed by immunofluorescence using anti-Myc and anti-HA antibodies to compare the subcellular localization of STAU2-tagged proteins. Both fusion proteins are cytoplasmic and mostly excluded from the nucleus, as expected. DAPI was used to stain the nucleus. **b** hTERT-RPE1 cells were infected with viruses expressing an empty vector or STAU2^52^-BioID2-HA. Two days post selection, cells were incubated in the absence (−) or presence (+) of 50 μM biotin for 16 h. Biotinylated proteins were visualized by Western blotting using streptavidin-coupled HRP. **c** 60 proteins with the best interaction probability scores (decreasing fold-change from top to bottom) are listed along with a heat map (right) representing the average spectral counts of STAU2 interactors. **d** Pie chart of the percentage of STAU2 interactors associated with different biological processes. Percentages represent the number of specific interactors in each molecular process/total number of specific interactors

As a means to identify the pathway(s) that benefits from STAU2 stabilization/degradation, we used BioID2, a genome wide approach that identifies proteins in close proximity to STAU2 (within a range of 10 nm). hTERT-RPE1 cells were infected with a virus expressing either STAU2^52^-biotinylase-HA or STAU2^52^-myc (as control) fusion proteins. We first showed by fluorescence microscopy (Fig. 3a) that the two fusion proteins localized in the cytoplasm as expected from the known distribution of STAU2 in these cells. Then, we confirmed that, in the presence of biotin, STAU2^52^-biotinylase-HA fusion protein can covalently add biotin on proteins (Fig. 3b). Then, we incubated cells with biotin for 16 h. Cells were collected, and biotinylated proteins were pulled down with streptavidin-coupled magnetic beads. Labeled proteins were identified by mass spectrometry (Supp Table S1). Interaction probability was analyzed with SAINT (Significance Analysis of INTeractome) [27] and potential off-targets were rejected using CRAPome (Contaminant Repository for Affinity Purification) [28]. We identified 325 peptides using a score of 0.7 or better (Supp Table S2). Known STAU2 interactors were found in the list of biotinylated proteins, including UPF1 [16], STAU1 [29], CDK1 [30], RSL1D1 [31], ZFR [32], and PABPC1 [33], confirming the efficacy and specificity of the assay. Proteins showing the highest enrichment (fold-changes) are presented along with a heat map representing their average spectral counts (Fig. 3c). In addition, the enriched GO terms are related to known STAU2 functions, such as translation, RNA metabolism, RNA localization, ribonucleoprotein complex biogenesis, RNA decay, splicing and stress granule formation (Supp Table S3). It is thus likely that a major consequence of STAU2 degradation will be on the posttranscriptional regulation of its targeted mRNAs. Interestingly, in addition to proteins of the posttranscriptional pathways, this approach also identified numerous proteins localized in the nucleolus including proteins involved in ribosome biogenesis, response to DNA damage, and DNA replication (Fig. 3d). STAU2-biotinylase often labeled all the proteins found within specific complexes indicating that STAU2 is in close proximity to functional complexes and not only to single proteins. For examples, not only the screen identified PRKDC (DNA-PK) but also its cofactors XRCC5 (KU80) and XRCC6 (KU70) that are recruited together at DNA damage foci [34] and all the components of the HEMIN1-DNA-PK-paraspeckel ribonucleoprotein complex [35]. Strikingly, a large percentage of the labeled proteins involved in DNA replication and/or DNA repair are linked to the CHK1 pathway (Supp Table S4), suggesting that STAU2 expression could also be modulated by this pathway. CHK1 is known to be involved in cell cycle checkpoints, DNA damage repair and DNA replication in unstressed cells [36].

### STAU2 protein level is regulated by the CHK1 pathway

**Fig. 4:**
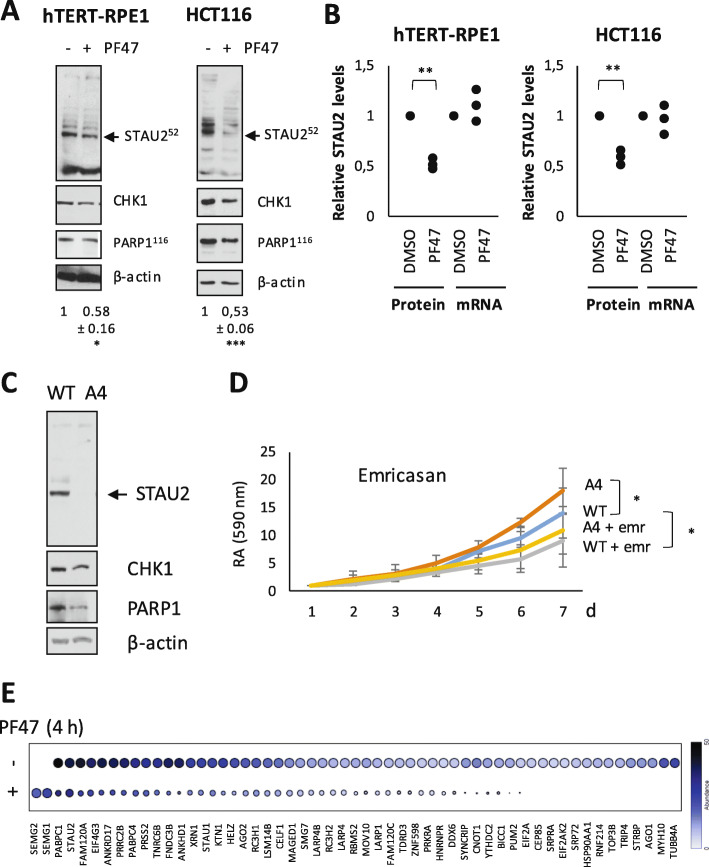
CHK1 inhibition causes a decrease in the steady-state levels of STAU2 protein. **a** hTERT-RPE1 and HCT116 cells were incubated in the presence of the CHK1 inhibitor PF47 (20 μM) for 8.5 h. Cell extracts were analyzed by Western blotting. The vehicle DMSO was used as control and β-actin as a loading control. PARP1 cleavage was used as a measure of apoptosis. Quantification of STAU2 protein levels is indicated below the blots. Western blots are representative of at least three independently performed experiments that gave similar results. **b** Cells were incubated in the presence of CHK1 inhibitor (PF47 20 μM) for 6.5 h. STAU2 protein expression was analyzed by western blotting, while STAU2 mRNA levels were quantified by RT-qPCR. The ratio of STAU2 (protein or mRNA) on actin (protein or mRNA, respectively) in DMSO-treated cells was fixed to 1. *n* = 3. ** *p*-value ≤0.01. One sample *t*-test. **c** WT and STAU2-KO A4 hTERT-RPE1 cells were analyzed by Western blotting for expression of STAU2, CHK1 and PARP1. Actin was used as a loading control. **d** WT and STAU2-KO A4 hTERT-RPE1 cells were treated with the pan-caspase inhibitor emricasan. Cell proliferation using growth curve assays was quantified every day using the crystal violet retention assay. Statistic: Dunnett’s multiple comparisons (**e**) Dotplot representation of protein abundance in proximity to STAU2 in the presence or absence of the CHK1 inhibitor PF47. Color range indicates peptide abundance and the size of circles their relative abundance

To determine if STAU2 is a downstream factor in the CHK1 pathway as suggested by the BioID2 assay, we treated the non-transformed hTERT-RPE1 and the cancer HCT116 cells with the CHK1 inhibitor PF-477736 (PF47) for 8 h (Fig. 4a). Our results indicated that STAU2 steady-state level significantly decreased following CHK1 inhibition. As control, we showed that the amount of CHK1 protein was downregulated in PF47-treated cells compared to untreated cells. Inhibition of CHK1 by two additional inhibitors (iCHK1 and CHIR124) also resulted in STAU2 depletion (Supp Fig. S4A), ruling out an off-target effect. Interestingly, the decrease of STAU2 protein levels coincided with a cleavage of PARP1, suggesting that CHK1 inhibition could activate caspases. To further eliminate putative off-target effect, we also treated cells with lower inhibitor concentrations (1 μM vs 20 μM) using longer incubation times. hTERT-RPE1 and HCT116 cells were incubated in the presence of CHK1 inhibitor PF47 for 48 hours, and STAU2 protein levels were quantified by Western blotting (Supp Fig. 4B). As observed above, inhibition of CHK1 with lower inhibitor concentrations still caused a decrease in STAU2 protein levels. To study the mechanism of downregulation, STAU2 protein and mRNA levels were quantified by Western blotting and RT-qPCR (Fig. 4b), respectively. Our results indicated that STAU2 steady-state level significantly decreased following CHK1 inhibition while mRNA levels were stable. Therefore, the decrease in STAU2 protein levels did not rely on transcriptional downregulation. These results indicate that STAU2 steady-state protein level is regulated by the CHK1 pathway, likely through an active mechanism of protein degradation. To determine if STAU2 is part of a feedback loop with CHK1, we quantified the amount of CHK1 protein in WT and STAU2-KO hTERT-RPE1 cells. Western blotting experiments indicated that the amount of CHK1 was decreased in STAU2-KO cell line (Fig. 4c), suggesting that STAU2 expression may have a positive feedback effect on CHK1 expression or activity.

It is well known that CHK1 activation activates checkpoint controls, and therefore its inactivation may be involved in the enhanced cell growth observed in STAU2-depleted cells. To determine if caspase activation observed in STAU2-depleted cells may also be involved in cell growth, growth curve (Fig. 4d) and colony formation (Supp Fig. S5) assays were performed in the absence or presence of the pan-caspase inhibitor emricasan. Our results showed that caspase inhibition slightly reduced cell proliferation, suggesting that the activation of caspases observed in STAU2-KO cells might be beneficial for cell growth.

### Inhibition of the CHK1 pathway dissociates STAU2 from RNA metabolism

To determine the immediate consequence of CHK1 inhibition on STAU2 proximal interactome, we identified proteins in proximity to STAU2 during the first 4 h following inhibition of CHK1 by PF47 using the Turbo-biotinylase. In contrast to BioID2 which necessitates labeling with biotin for 16–24 h, the high activity of turboID allows short incubation times and thus higher temporal resolution of protein interaction. A virus expressing STAU2^52^/turbo-biotinylase was infected in hTERT-RPE1 cells and the activity of the fusion protein was induced by addition of biotin in the presence or absence of the CHK1 inhibitor PF47. Biotinylated proteins were isolated 4 h later and analyzed by mass spectrometry (Supp Table S5). In the absence of PF47, STAU2 is in close proximity to proteins involved in translation and RNA metabolism as described above (Supp Tables S6, S7). However, in the presence of PF47, 57 of the previously identified proximity partners were absent or less abundant (Supp Table S8). Proteins showing the highest fold change are presented in Fig. 4e. Proteins enriched in GO terms related to translation, miRNA metabolic processes and mRNA catabolism were lost (Supp Table S9). Interestingly, this change in STAU2 interactome occurs prior to STAU2 degradation, indicating that STAU2 functions are rapidly affected in response to CHK1 inhibition, before its degradation. Interestingly, only two proteins (SMEG1 and SMEG2) increased their proximity to STAU2 in these conditions. The role of these proteins is poorly understood. They were nevertheless previously described as modulators of zinc-dependent proteases via their high capacity to binding zinc [37] and shown to co-immunoprecipitate with several proteins including proteins involved in checkpoint controls and DNA damage repair [38].

## Discussion

**Fig. 5:**
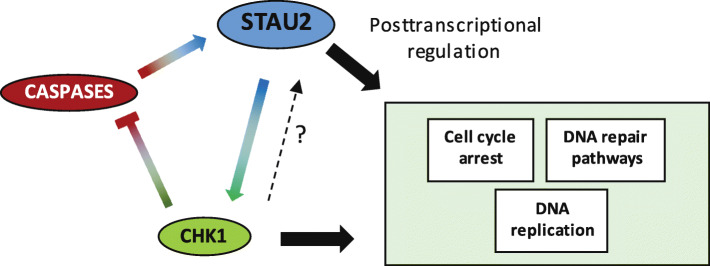
Schematic representation of the STAU2/CHK1 regulatory loop. CHK1 is well known for its role in cell cycle arrest and DNA repair following single-stranded DNA damage. In unstressed cells, CHK1 is involved in DNA replication, DNA repair and cell cycle arrest. Our results suggest that CHK1 positively controls the amount of STAU2 via inhibition of caspases. In turn, STAU2 expression positively regulates the amount of CHK1 protein, likely via the posttranscriptional regulation of CHK1 mRNA [10], linking STAU2 to cell cycle arrest and DNA repair. In addition to its role on CHK1 regulation, STAU2 may control the posttranscriptional regulation of multiple mRNAs coding for proteins involved in DNA repair, cell proliferation and DNA replication pathways. This figure was created with PowerPoint (Microsoft Office Professionnel Plus 2016)

In this paper, we investigated the consequence of STAU2 depletion in non-transformed and cancer cells. We show that STAU2 depletion accelerates cell cycle progression in non-transformed cells but not in cancer cells. Interestingly, under physiological conditions, the steady state level of STAU2 protein is controlled by caspases and by the activity of the CHK1 pathway (Fig. 5). This loop likely contributes to the fine-tuning of cell proliferation in changing cell environment. The regulation of STAU2 at the protein level is in addition to that already observed at the transcriptional level by the transcription factor E2F1 [25]. Regulation of STAU2 by the E2F1 and CHK1 pathways that induce and facilitate S phase progression and DNA surveillance suggests a role for STAU2 in pathways that merge with mechanisms of DNA replication and/or DNA maintenance during S phase.

### STAU2 regulates cell growth

The mechanism by which STAU2 depletion facilitates cell growth is not clear. STAU2, as an RNA-binding protein involved in the posttranscriptional regulation of gene expression, was previously shown to bind mRNAs coding for proteins involved in cell cycle regulation and cell cycle proliferation, including CHK1 mRNA [10]. We hypothesize that in unstressed cells, STAU2 expression may stabilize CHK1 mRNA, thus the amount of CHK1 protein, causing activation of checkpoint controls. Upon STAU2 depletion, the amount of the CHK1 protein decreased (Fig. 4c) likely via altered posttranscriptional regulation of its mRNA. The physiological decrease of CHK1 activity that releases checkpoint controls may be part of the mechanism that leads to enhanced cell proliferation upon STAU2 depletion. This mechanism contributes to the fine-tuning of both STAU2 and CHK1 expression.

Similarly, STAU2 was shown to bind 26 mRNAs involved in the TGF-β pathway, including the central protein TGF-β receptor I [10]. It was recently reported that dysregulation of the TGF-β pathway in RPE1 cells leads to cell cycle dysregulation and tumoral transformation [39]. The phenotypes observed upon STAU2 depletion are indeed compatible with oncogenic transformation. Oncogenic transformation was previously observed when RNA-binding proteins inappropriately misregulated proto-oncogenes and/or tumor suppressor genes [3, 6, 7, 40, 41]. The hyper-proliferative phenotype that results from the misregulation of these RNA-binding proteins is often followed by an accumulation of DNA damage due to the loss of cell cycle checkpoints [42, 43]. Accumulation of DNA damages are indeed observed in STAU2-KO hTERT-RPE1 cells (Supp Fig. S6) and in RNAi-mediated STAU2 knockdown in HCT116 cells [25]. Decrease in the amount of CHK1 upon STAU2 depletion may be part of the mechanism that leads to increase DNA damages. Oncogenic transformation may also explain why tumor cells are not affected by STAU2 depletion. Transformed cell lines are known to be permanently stimulated by oncogenic stimulus [44, 45], and therefore additional oncogenic stimulus may not affect their proliferation.

### STAU2 protein is degraded by caspases and stabilized by the CHK1 pathway

Under physiological conditions, STAU2 protein level is decreased by caspase activity (Fig. 2) and stabilized by the CHK1 pathway (Fig. 4). As CHK1 activation inhibits caspase activity [46] and CHK1 inhibition increases PARP1 degradation via caspases (Fig. 4), it is tempting to propose that CHK1 indirectly controls STAU2 steady-state levels via the activation/inhibition of caspase activity (Fig. 5). It is known that the expression of STAU2 [25] and of CHK1 [47] is upregulated at the G_1_/S phase of the cell cycle by the transcription factor E2F1. Our results now support the hypothesis that the amounts of STAU2 and CHK1 proteins are then positively regulated by CHK1-mediated caspase inhibition and by STAU2-mediated posttranscriptional regulation of CHK1 mRNA, respectively. Although well characterized for their roles in response to massive DNA damages and induction of apoptosis [48–52], caspases also play other important roles in unstressed cells related to cell proliferation, differentiation and cellular reprogramming [53–55]. In turn, CHK1 is involved in cell cycle control, especially during the S phase, where it is required for firing late origin of DNA replication [56]. CHK1 regulates checkpoint controls and is essential for cell survival in dividing cells [57] and its inhibition induces an accumulation of DNA damage and apoptosis [58, 59].

Alternatively, CHK1 may control STAU2 stability via activation of downstream kinases as previously described for the CHK1-mediated control of the RNA-binding protein HuR via the regulation of CDK1 [60]. Interestingly, STAU2 is a target of CDK1 [30] and CDK1 was found in the list of proteins in proximity to STAU2 (Supp Table S1). In contrast, we have no evidence that STAU2 is a direct target of CHK1. CHK1 was not found in the list of proteins in proximity to STAU2 and, using an in-vitro kinase assay, we did not observe phosphorylation of STAU2 by a purified CHK1 kinase (data not shown).

### STAU2 is in close proximity to proteins involved in RNA posttranscriptional regulation and proteins of the nucleolus

Degradation/stabilization of STAU2 and/or modulation of STAU2 functions via proximal partners should influence the pathway(s) in which STAU2 is involved. The BioID2 and TurboID experiments that detect proteins in close proximity to STAU2 link STAU2 to its well recognized functions in RNA posttranscriptional regulation. Indeed, STAU2 is well known for its roles in translation [15], RNA localization [9, 12, 15], splicing [14], mRNA decay [16], and stress granule formation [61]. It is likely that STAU2 regulation by caspases and/or the CHK1 pathway fine tunes the expression of mRNA regulons, resulting in well-ordered cell proliferation.

Interestingly, these genome-wide experiments also identify many proteins that can be found in the nucleolus. The nucleolus is well known for its role in ribosome biogenesis and for its involvement in non-ribosomal ribonucleoprotein complex formation [62]. Labeling of proteins found in the nucleolus compartment is consistent with earlier observation showing that STAU2 can migrate in the nucleolus [11]. The role of STAU2 in the nucleolus is unclear but it was proposed that STAU2 may assemble ribonucleoprotein complexes in the nucleolus [63]. The presence of ribosomes in STAU2-containing ribonucleoprotein complexes [9] further suggests a functional link between ribosome biogenesis and RNP formation in the nucleolus. STAU2 indeed co-immunoprecipitates with the ribosome biogenesis factor RSL1D1 [31]. The nucleolus might function as a checkpoint to verify the potential functional integrity of RNP and RNP-ribosome complexes or to tightly regulate their activity and/or release. Depletion of STAU2 may thus cause the formation of less-functional ribonucleoprotein complexes that could be released ahead of time or in inappropriate cellular compartments, impairing cell proliferation.

Other proteins in proximity to STAU2 are members of the DNA damage response. Most of these proteins can be found in the nucleoplasm and in the nucleolus and, therefore, could be non-specifically labeled via their co-localization with STAU2 in the nucleolus. However, STAU2 depletion causes an accumulation of DNA damages and therefore could be somehow involved in processes of DNA repair. Several processes could link STAU2 to DNA repair pathways. First, rDNA genes are the most transcribed genes and thus collisions between the transcription machinery and the replication fork are frequent [64, 65]. Consistently, several proteins involved in DNA replication are found in proximity to STAU2. The clashes between the machineries lead to replication fork stalling and single-stranded breaks [62]. This, in turn, activates the CHK1 kinase pathway [66–68]. In addition, R-Loops forms between nascent mRNAs and template DNA strands leading to genomic instability [69–71]. Remarkably, dysregulation of post-transcriptional processes increases R-loops formation [69, 72]. Depletion of STAU2 may thus facilitate the formation of R-loops whereas STAU2 stabilization upon CHK1 activation may help to attenuate the effects of R-loop formation. Alternatively, several studies have revealed the importance of lncRNAs in the DNA damage repair processes [73]. lncRNAs act as scaffolds for DNA repair protein recruitment. For example, lncRNA LINP1 participates in DNA-PK recruitment to DSB [74] and lncRNA NEAT1 is needed for the formation of DNA-PK/HEXIM1 paraspeckle complexes [35]. In other cases, lncRNAs play a role in chromatin modification, which is an indispensable step for the DNA repair process [75]. It is conceivable that STAU2 may be required to localize lncRNAs at the DNA damage sites to support the repair process.

## Conlusions

In this paper, we describe a complex network of proteins in proximity to STAU2 that influences the roles of STAU2 in mRNA translation and RNA metabolism. This study also opens new research fields in post-transcriptional regulation of gene expression related to the fine-tuning of cell proliferation, ribosome biogenesis and DNA damage response.

## Methods

### Plasmids and cloning strategies

Plasmids coding for STAU2^52^-FLAG_3_ and STAU2^59^-FLAG_3_ were previously described [25]. To generate the retroviral pMSCVpuro-STAU2^52^-BioID-HA construction, STAU2^52^ was PCR-amplified using Phusion polymerase (NEB) and STAU2^52^-FLAG_3_ as template (FW: 5′ TAAGCAGCTAGCATGCTTCAAATAAATCAGATGTTCT 3′; RV: 5′ TGCTTAACCGGTCTACCTGAAAGCCTTGAATCCT 3′). The PCR product was digested by NheI and AgeI and cloned into pcDNA3.1 BioID2-HA vector [MCS-BioID2-HA was a gift from Kyle Roux (Addgene plasmid # 74224; http://n2t.net/addgene:74224; RRID:Addgene_74224)] [76]. The resulting product was PCR-amplified and cloned into retroviral pMSCV puromycin vector after EcoRV digestion (FW: 5′.

GCTAGCATGCTTCAAATAAAT 3; RV: 5′ GTTTAAACTTAAGCTTCTATGCG 3′). Three copies of the myc sequence were introduced at the NotI site.

To generate the retroviral pMSCVpuro-STAU2^52^-TurboID-HA and pMSCVpuro-YFP-TurboID-HA vectors, STAU2^52^-FLAG and YFP were PCR-amplified using Phusion polymerase (NEB) and oligonucleotide primers (STAU2: FW: 5′ TAAGCAGCGGCCGCATGCTTCAAATAAATCAGATGTTCTCAG 3′; RV: 5’TGCTTAGCTAGCGGATCCGAATTCGAATCCGGAGACGTACGACCGGTCTACCTGAAAGCCTTGAATCCTTG 3′) (YFP: FW: 5′ TAAGCAGCGGCCGCGCCACCATGGTGAGCAAG 3′; RV: 5′ TGCTTAGCTAGCGGATCCGAATTCGAATCCGGAGACGTACGACCGGTCCTTGTACAGCTCGTCCATGC 3′). The PCR products were digested by NotI and NheI and cloned into pcDNA3.1 TurboID-HA vector [V5-TurboID-NES_pCDNA3 was a gift from Alice Ting (Addgene plasmid # 107169; http://n2t.net/addgene:107169; RRID: Addgene_107169)]. The resulting products were PCR-amplified and cloned into retroviral pMSCV puromycin vector after NotI digestion (STAU2: FW: 5′ TAAGCAGCGGCCGCGCCACCATGCTTCAAATAAATCAG 3′; RV: 5′ TGCTTAGCGGCCGCCTATGCGTAATCCGGTACATCGTAAGGGTATCCCTTTTCGGCAGACCGCAG 3′) (YFP: FW: 5′ TAAGCAGCGGCCGCGCCACCATGGTGAGCAAG 3′; RV: 5′ TGCTTAGCGGCCGCTTACTATGCGTAATCCGGTACATCGTAAGGGTATCCGTCCAGGGTCAGGCGCTC 3′).

### Generation of STAU2-KO hTERT-RPE1 cell lines and genomic sequencing

hTERT-RPE1 cells were transfected with a plasmid coding for GFP, Cas9, and a sgRNA targeting exon 6 of the *STAU2* gene (Horizon Discovery). Forty-eight hours post-transfection, GFP positive cells were sorted by FACS and plated into a 10 cm dish. Forty-eight hours after the first sorting, cells were once again isolated by FACS, and individual cells were grown into 96-well plates until colonies formed. The loss of STAU2 expression was monitored by dot blotting using anti-STAU2 antibody. Genomic DNA of several clones was isolated (Genomic DNA Miniprep Kit, Bio Basic) and PCR-amplified with specifics primers flanking exon 6 of the *STAU2* gene (FW: 5′ AGCAGAATTCTTGGATAGGATAGAACAGAATTTGG 3′; RV: 5′ ATTAGGATCCACACACATAGCAGACAACATAAC 3′). PCR products were cloned into a pBluescript SK (+) vector (Stratagene) and sequenced (Sanger Sequencing Services Genome Québec).

### Antibodies and reagent

Primary Antibodies against CHK1 (2G1D5), PARP1 (46D11), H2AX (D17A3), and ɣH2AX (20E3) were purchased from Cell Signaling; against GAPDH (0411), HA (12CA5) and Myc (9E10) were purchased from Santa Cruz Biotechnology. Anti-STAU2 (HPA019155), anti-β-Actin (A5441), anti-Flag (F3165), and HRP-Streptavidin were obtained from Sigma. All primary antibodies were used at 1:1000 dilution. MG132 (C2211), iCHK1 (681637) and DMSO were purchased from Millipore-Sigma; ZVAD-FMK (S7023), Emricasan (S7775), PF47 (PF-477736, S2904), and CHIR124 (S2683) were obtained from Selleckchem.

### Cell culture

hTERT-RPE1 (non-transformed retinal pigment epithelial cell line immortalized with hTERT telomerase), HCT116 (colorectal carcinoma cell line) and HeLa (cervical cancer cell line) cells were cultured in Dulbecco modified Eagle’s medium (DMEM, Wisent) supplemented with 10% fetal bovine serum (Wisent), 100 μg/ml streptomycin and 100 units/ml penicillin (Wisent) under 5% CO2 atmosphere.

### DNA transfection and infection

For transient expression, cells were transfected with lipofectamine 2000 (Invitrogen) or Mirus X2 (Mirus Bio. LLC) at approximatively 60% confluency. For infections, Phoenix cells were transfected at approximatively 50% confluency using lipofectamine 2000, with 10 μg of retroviral plasmids (pMSCV-puromycin) and 5 μg of packaging plasmid. Virus-containing supernatant were collected, filtered (0.45 μm), and added to target cells (HCT116, hTERT-RPE1) with polybrene (8 μg/ml). Infected cells were selected on puromycin (HCT116, 2 μg/ml; hTERT-RPE1, 5 μg/ml).

### Protein electrophoresis and Western blot analysis

Total cell extracts were prepared in lysis buffer (50 mM Tris–HCl pH 7.5, 15 mM EDTA, 0.5% Triton X-100, 100 mM NaCl, 1 mM dithiothreitol [DTT] and a protease inhibitor cocktail [Roche]), resolved by SDS–polyacrylamide gel electrophoresis and transferred to nitrocellulose membrane. Membranes were blocked in 5% milk at room temperature and incubated with primary antibodies overnight at 4 °C prior to incubation with HRP-conjugated secondary antibody [polyclonal anti-mouse (1/3000), Dako: P0447; polyclonal anti-rabbit (1/5000), Dako: P0448] for 1 h at room temperature. Membranes were processed using Perkin Elmer Western Lightning Plus-ECL. Data were collected either on X-ray films (Fujifilm) or with the ChemiDoc MP Imaging System (Bio-Rad Laboratories). Western blot signals were quantified with the ImageLab (Bio-Rad Laboratories) software or with ImageJ software (NIH.gov). For the detection of biotinylated proteins by western blot, membranes were blocked in PBS-BSA 5% and incubated with HRP-streptavidin 0.3% in PBS-BSA 3% for 45 min at room temperature.

### RNA isolation and RT-qPCR

Total RNA was isolated from cell extracts using the Geneaid extraction kit. Purified RNA was resuspended in 40 μl of water and digested with DNase using the TURBO DNA-free kit (Ambion). Reverse transcription reactions were done with 1 μg of RNA using RevertAid H Minus First Strand cDNA Synthesis kit (Thermo Scientific. qPCR was performed with Luna® Universal qPCR Master Mix (NEB) on a LightCycler 96 instrument (Roche). Samples were run in triplicates and normalized to actin expression.

### Cell growth assays

To determine cell proliferation rates, cells were plated at the same density (Growth curve assay: HeLa: 5000 cells, hTERT-RPE1: 8000 cells and HCT116 5000 cells. Colony assay: HeLa 15,000 cells, hTERT-RPE1: 15000 or 25,000 cells, HCT116 15,000 cells) and allowed to grow for 9 days. Cell proliferation was quantified using a crystal violet retention assay (39). For growth curve assay, cells were harvested every day and the number of cells was counted with an automated hemacytometer.

### BioID2 and TurboID sample preparations

One (TurboID) or two (BioID2) biological replicates were processed independently as described [77]. Briefly, stable cell line expressing STAU2^52^-BioID-HA, STAU2^52^-TurboID-HA, or YFP-TurboID-HA were generated by retroviral infection. After puromycin selection, cells were grown to 70% confluency and were incubated in the presence of 50 μM biotin for 16 h (BioID2) or 4 h (TurboID) and lysed in 700 μL RIPA lysis buffer (50 mM Tris pH 8, 150 mM NaCl, 0.1% SDS, 0.5% sodium deoxycholate, 1% Triton X-100, 1× protease inhibitor cocktail, Sigma-Aldrich). Cell extracts were sonicated and biotinylated proteins pulled down with 250 μL streptavidin-coated magnetic beads (Dynabeads MyOne Streptavidin T1). Beads were then washed twice with 1 mL of RIPA lysis buffer, once with 1 mL of 1 M KCl, once with 1 mL of 0.1 M Na2CO3, once with 1 mL of 2 M urea in 10 mM Tris-HCl (pH 8.0), and three time with 1 mL RIPA lysis buffer. Beads were then resuspended in 100 μL 50 mM ammonium bicarbonate solution.

### Protein digestion and LC-MS/MS

Proteins were identified at the proteomic core facility for LC-MS/MS analysis. Proteins were digested on beads with 1 μg Sequencing Grade Modified Trypsin (Promega) overnight at 37 °C. Peptides in the supernatant were reduced with 9 mM dithiothreitol at 37 °C for 30 min and alkylated with 17 mM iodoacetamide at room temperature for 20 min. After desalting, peptides were eluted in 10% ammonium hydroxide/90% methanol (v/v) and resuspended in 5% FA. Peptides were loaded into a 75 μm i.d. × 150 mm Self-Pack C18 column installed in the Easy-nLC II system (Proxeon Biosystems). The buffers used for chromatography were 0.2% formic acid (buffer A) and 90% acetonitrile/0.2% formic acid (buffer B). Peptides were eluted with a two slope gradient at a flowrate of 250 nL/min. Solvent B first increased from 1 to 35% in 105 min and then from 35 to 84% B in 15 min. The HPLC system was coupled to a Q Exactive mass spectrometer (Thermo Scientific) through a Nanospray Flex Ion Source. Nanospray and S-lens voltages were set to 1.3–1.8 kV and 50 V, respectively. Capillary temperature was set to 225 °C. Full scan MS survey spectra (m/z 360–2000) in profile mode were acquired in the Orbitrap with a resolution of 70,000. The 16 most intense peptide ions were fragmented in the collision cell and MS/MS spectra were analyzed in the Orbitrap.

### Protein identification and enrichment analysis

The peak list files were generated with Proteome Discoverer (version 2.1) using the following parameters: minimum mass set to 500 Da, maximum mass set to 6000 Da, no grouping of MS/MS spectra, precursor charge set to auto, and minimum number of fragment ions set to 5. Protein database searching was performed with Mascot 2.6 (Matrix Science) against the Uniprot human protein database (May 16th, 2018). The mass tolerances for precursor and fragment ions were set to 10 ppm and 0.6 Da, respectively. Trypsin was used as the enzyme allowing for up to 1 missed cleavage. Cysteine carbamidomethylation was specified as a fixed modification, and methionine oxidation as variable modifications. Data interpretation was performed using Scaffold (version 4.8.9). Protein lists were submitted to the CRAPome software to calculate SAINT score and the fold change scores (FC-A, FC-B). P Proteins with SAINT probability (SP) greater than 0.7 were selected. REPRINT tools were used to generate visual analysis of our mass spectrometry data.

### Epifluorescence microscopy

Cells were seeded onto 12 × 12 mm glass coverslips in 6-well plates and incubated overnight at 37 °C. Cells were fixed in 4% PFA for 15 min at room temperature and permeabilized in 10 mM Tris (pH 7.5), 0.5% Triton-X-100 (v/v) and 0.1% BSA (w/v) for 10 min. Cells were then stained with anti-myc (1600) or anti-HA: (1500) for 1 h at room temperature. Secondary antibody (Alexa Fluor 488 goat) was added for 1 h at room temperature. Coverslips were washed once with PBS and incubated for 10 min with PBS containing DAPI (0.5 μg/ml) at room temperature. Coverslips were mounted using Dako mounting medium (Dako Faramount Aqueous Mounting Medium; S3025). Images were acquired with an ECLIPSE TE2000U microscope using 100X/1.40 Oil objective and chroma filter 41,001 (GFP) or chroma filter 31,000 (DAPI) (NIKON). Images processing was performed using ImageJ software. The data are representative of at least 15 fields of view.

## Supplementary Information

**Additional file 1: Figure S1.** Knockout of STAU2 in the nontransformed hTERT-RPE1 cell line by the CRISPR/Cas9 technique. (A) Schematic representation of the *STAU2* gene. Exons are indicated as well as the differential splicing of exon 5 and the differential choice of stop codons that contribute to the expression of several protein isoforms. The 3’end sequence of STAU2 exon 6 of the human genome (WT) is shown. The target sequence of the RNA guide used for CRISPR gene editing is underlined. The sequences of two different CRISPR/Cas9-derived STAU2-KO clones are shown below. Dashed lines indicate deleted nucleotides; the premature stop codons are indicated. (B) hTERT-RPE1 cells were transfected with plasmids expressing a Cas9/sgRNA complex targeting exon 6 of the *STAU2* gene. Colonies grown from single cells were screened for STAU2 protein expression by dot blotting. **(C)** Four different STAU2-KO clones and one CRISPR-derived clones that express STAU2 (I2) were analyzed by RT-qPCR for STAU2 expression. The ratio of STAU2 mRNAs on actin mRNA in CRISPR-control cells (I2) cells was arbitrary fixed to 1. The graph represents the means and standard deviation of three independently performed experiments. *** *p-*value ≤0.001; ** *p-*value ≤0.01. One sample *t*-test. Note that clones A4, B5 and C6 were originally named F3, F4 and G4, respectively.

**Additional file 2: Figure S2.** STAU2 depletion facilitates cell growth. Representative images of the colony growth assays (quantified in Fig. 1). The pictures are representative of three independently performed experiments. (A) WT and STAU2-KO (A4, B5) hTERT-RPE1 cells. (B,C) hTERT-RPE1 (B) and HeLa (C) cells infected with non-targeting shRNA (shNT) or shRNA against STAU2 (shSTAU2). (D) CRISPR-infected cells that still express STAU2 (Ctrl) and STAU2-KO (STAU2-KO) HCT116 cells.

**Additional file 3: Figure S3.** Knockout of STAU2 in HCT116 cancer cells. (A) Schematic representation of the *STAU2* gene, including the 5’end sequence of STAU2 exon 6 of the human genome and the position of the RNA guide RNA (underlined). (B) Western blot of CRISPR-transfected HCT116 cells grown from single cells to monitor STAU2 protein expression. 35% of the selected clones were negative for STAU2 expression.

**Additional file 4: Figure S4.** CHK1 inhibition causes a decrease in the steady-state levels of STAU2 protein. (A) HCT116 cells were incubated in the presence of CHK1 inhibitors (PF47 20 μM, iCHK1 20 μM for 8.5 h and CHIR124 200 nM for 24 h). (B) hTERT-RPE1 and HCT116 cells were incubated in the presence of low concentration of the CHK1 inhibitor PF47 (1 μM) for 48 h. Cell extracts were analyzed by Western blotting. The vehicle DMSO was used as control and β-actin as a loading control. PARP1 cleavage was used as a measure of apoptosis. Quantification of STAU2 protein levels is indicated below the blots. Western blots are representative of at least three independently performed experiments that gave similar results.

**Additional file 5: Figure S5.** Caspases inhibition alters cell growth. WT and STAU2-KO A4 hTERT-RPE1 cells were treated with the pan-caspase inhibitor emricasan and allow to grow for 7 days. Colony growth assays were used to monitor cell proliferation. Left: representative growth of cells plated in triplicates. Right: Quantification of cell growth from three independently performed experiments. The relative growth of wild-type cells was arbitrary fixed to 1. ** *p*-value ≤0.01; * *p*-value ≤0.05. One sample *t*-test.

**Additional file 6: Figure S6.** STAU2 depleted cells accumulate DNA damages. (A) Protein extracts isolated from WT and STAU2-KO hTERT-RPE1 cells were analyzed by Western Blotting for STAU2 and H2AX expression. Phosphorylated H2AX (γH2AX), a marker of DNA damages, was also revealed. The western blot is representative of three independently performed experiments. (B) Quantification of ɣH2AX protein levels in four different hTERT-RPE1 STAU2-KO cells compared to WT cells. Protein quantification represents the means and standard deviation of three independently performed experiments. The ratio of ɣH2AX on H2AX in wild type (WT) cells was arbitrary fixed to 1. ** *p-*value ≤0.01. One sample *t*-test.

**Additional file 7: Figure S7.** Uncropped images of immunoblots used in figures.

**Additional file 8: Table S1.** List of all biotinylated proteins in the BioID2 assay.

**Additional file 9: Table S2.** List of STAU2 interactors with an interaction probability score of 0.7 or better as calculated with the SAINT and CRAPome softwares.

**Additional file 10: Table S3.** Gene ontology (GO) of STAU2 interactors in hTERT-RPE1 cells. List of significant shared GO terms that are over-represented in BioID2, using Metascape (A Gene Annotation & Analysis Resources [78]).

**Additional file 11: Table S4.** List of STAU2 interactors involved in DNA repair and/or replication pathways. Proteins linked to the CHK1 pathway are indicated.

**Additional file 12: Table S5.** List of all biotinylated peptides in the TurboID assay. The TurboID assay was performed in standard conditions or in the presence of the CHK1 inhibitor PF47.

**Additional file 13: Table S6.** List of STAU2 interactors in the TurboID assay with an interaction probability score of 0.7 or better, using the SAINT and CRAPome softwares.

**Additional file 14: Table S7.** Gene ontology (GO) of STAU2 interactors in hTERT-RPE1 cells. List of significant shared GO terms that are over-represented in TurboID, using Metascape (A Gene Annotation & Analysis Resources [78]).

**Additional file 15: Table S8.** List of STAU2 interactors that are lost in PF47 conditions.

**Additional file 16: Table S9.** Gene ontology (GO) of STAU2 interactors that show reduced proximity to STAU2 in hTERT-RPE1 cells treated with the CHK1 inhibitor PF47 compared to untreated cells. List of significant shared GO terms that are over-represented in TurboID, using Metascape (A Gene Annotation & Analysis Resources [78]).

## Data Availability

All data generated or analyzed during this study are included in this published article [and its supplementary information files].

## Abbreviations

STAU2: Staufen2
CRISPR: Clustered Regularly Interspaced Short Palindromic Repeats
CHK1: checkpoint kinase 1
DDR: DNA damage response
RBP: RNA-binding protein
KO: knockout
WT: wild type
shRNA: short hairpin RNA
GO: gene ontology
lncRNA: long non-coding RNA
CRAPome: Contaminant Repository for Affinity Purification
REPRINT: Resource for Evaluation of Protein Interaction Networks
SAINT: Significance Analysis of INTeractome
