# Supplementary figures and images for: STAU2 protein level is controlled by caspases and the CHK1 pathway and regulates cell cycle progression in the non-transformed hTERT-RPE1 cells
Source: BMC Mol Cell Biol. 2021 Mar 4;22:16. doi: 10.1186/s12860-021-00352-y (PMC7934504; doi:10.1186/s12860-021-00352-y)

Supplementary Figure S1

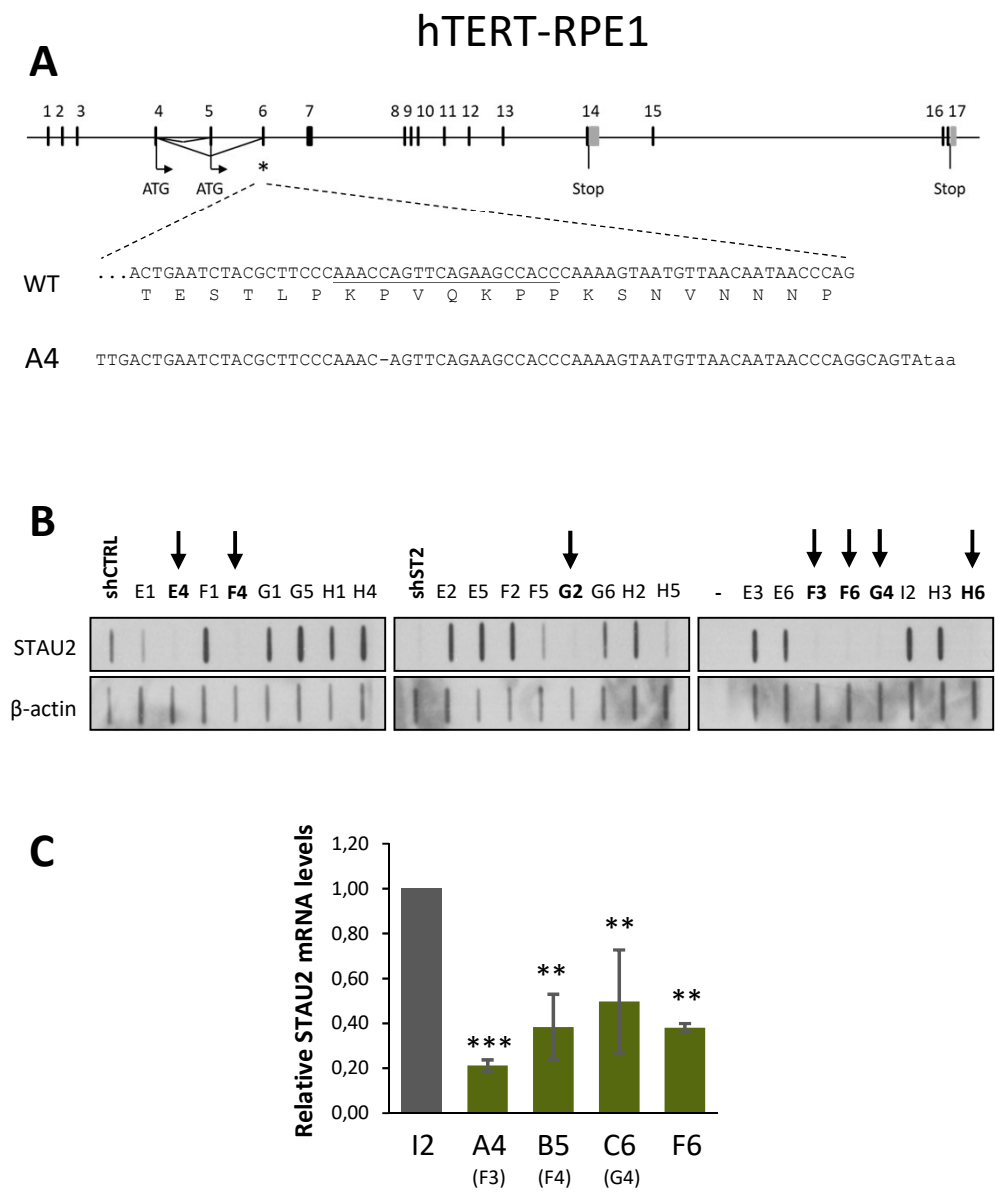

Supplement: Supplementary file 1 — Additional file 1: Figure S1. Knockout of STAU2 in the nontransformed hTERT-RPE1 cell line by the CRISPR/Cas9 technique. (A) Schematic representation of the STAU2 gene. Exons are indicated as well as the differential splicing of exon 5 and the differential choice of stop codons that contribute to the expression of several protein isoforms. The 3’end sequence of STAU2 exon 6 of the human genome (WT) is shown. The target sequence of the RNA guide used for CRISPR gene editing is underlined. The sequences of two different CRISPR/Cas9-derived STAU2-KO clones are shown below. Dashed lines indicate deleted nucleotides; the premature stop codons are indicated. (B) hTERT-RPE1 cells were transfected with plasmids expressing a Cas9/sgRNA complex targeting exon 6 of the STAU2 gene. Colonies grown from single cells were screened for STAU2 protein expression by dot blotting. (C) Four different STAU2-KO clones and one CRISPR-derived clones that express STAU2 (I2) were analyzed by RT-qPCR for STAU2 expression. The ratio of STAU2 mRNAs on actin mRNA in CRISPR-control cells (I2) cells was arbitrary fixed to 1. The graph represents the means and standard deviation of three independently performed experiments. *** p-value ≤0.001; ** p-value ≤0.01. One sample t-test. Note that clones A4, B5 and C6 were originally named F3, F4 and G4, respectively. [file 12860_2021_352_MOESM1_ESM.pdf]

Supplementary Figure S2

**A** hTERT-RPE1

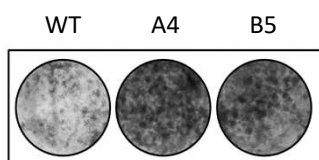

**B** hTERT-RPE1

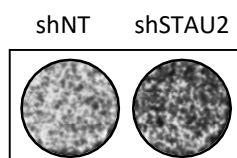

**C** HeLa

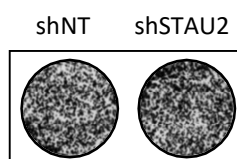

**D** HCT116

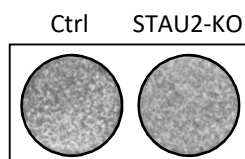

Supplement: Supplementary file 2 — Additional file 2: Figure S2. STAU2 depletion facilitates cell growth. Representative images of the colony growth assays (quantified in Fig. 1). The pictures are representative of three independently performed experiments. (A) WT and STAU2-KO (A4, B5) hTERT-RPE1 cells. (B,C) hTERT-RPE1 (B) and HeLa (C) cells infected with non-targeting shRNA (shNT) or shRNA against STAU2 (shSTAU2). (D) CRISPR-infected cells that still express STAU2 (Ctrl) and STAU2-KO (STAU2-KO) HCT116 cells. [file 12860_2021_352_MOESM2_ESM.pdf]

Supplementary Figure S3

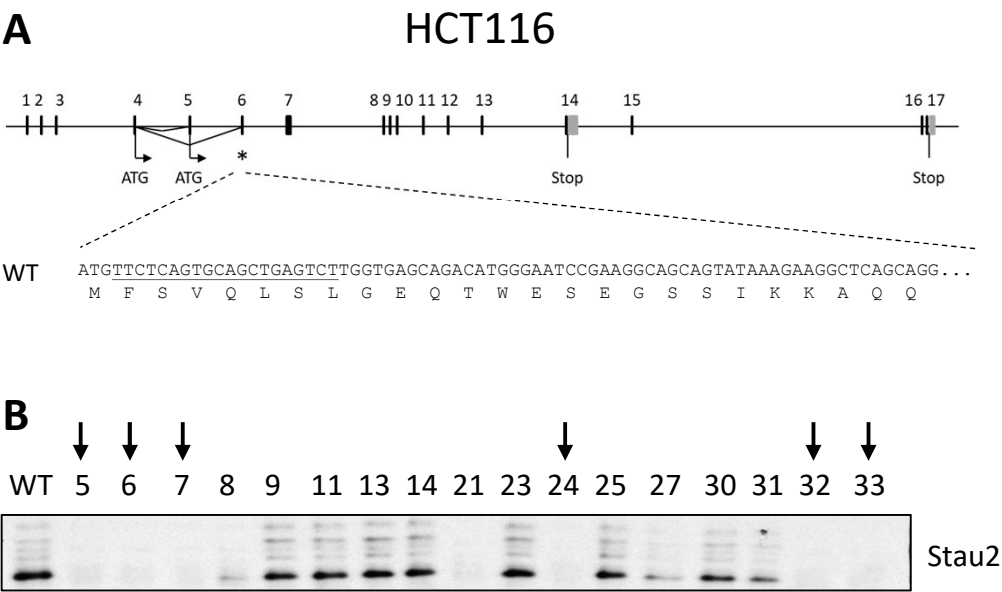

Supplement: Supplementary file 3 — Additional file 3: Figure S3. Knockout of STAU2 in HCT116 cancer cells. (A) Schematic representation of the STAU2 gene, including the 5’end sequence of STAU2 exon 6 of the human genome and the position of the RNA guide RNA (underlined). (B) Western blot of CRISPR-transfected HCT116 cells grown from single cells to monitor STAU2 protein expression. 35% of the selected clones were negative for STAU2 expression. [file 12860_2021_352_MOESM3_ESM.pdf]

Supplementary Figure S4

**A**

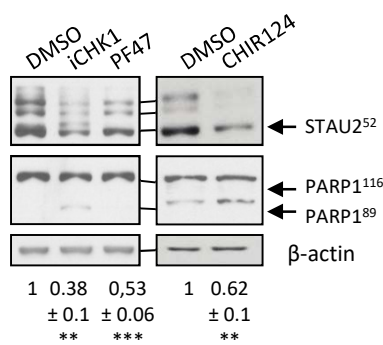

**B**

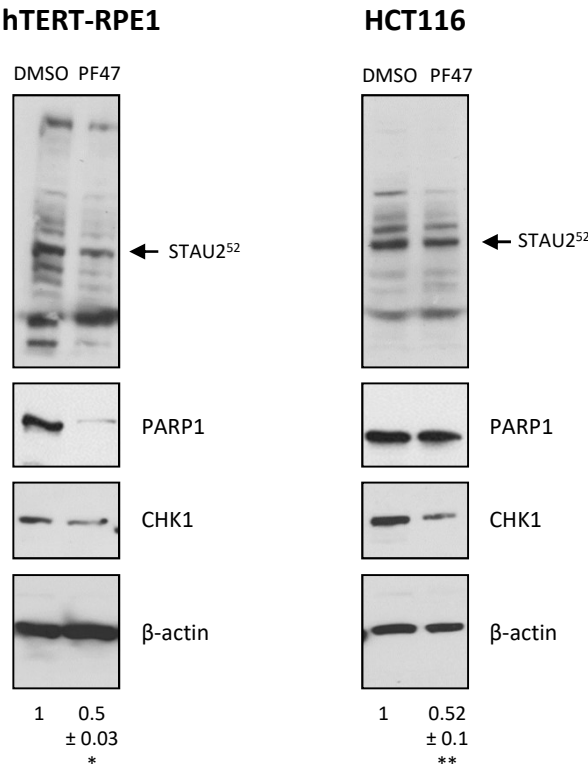

Supplement: Supplementary file 4 — Additional file 4: Figure S4. CHK1 inhibition causes a decrease in the steady-state levels of STAU2 protein. (A) HCT116 cells were incubated in the presence of CHK1 inhibitors (PF47 20 μM, iCHK1 20 μM for 8.5 h and CHIR124 200 nM for 24 h). (B) hTERT-RPE1 and HCT116 cells were incubated in the presence of low concentration of the CHK1 inhibitor PF47 (1 μM) for 48 h. Cell extracts were analyzed by Western blotting. The vehicle DMSO was used as control and β-actin as a loading control. PARP1 cleavage was used as a measure of apoptosis. Quantification of STAU2 protein levels is indicated below the blots. Western blots are representative of at least three independently performed experiments that gave similar results. [file 12860_2021_352_MOESM4_ESM.pdf]

Supplementary Figure S5

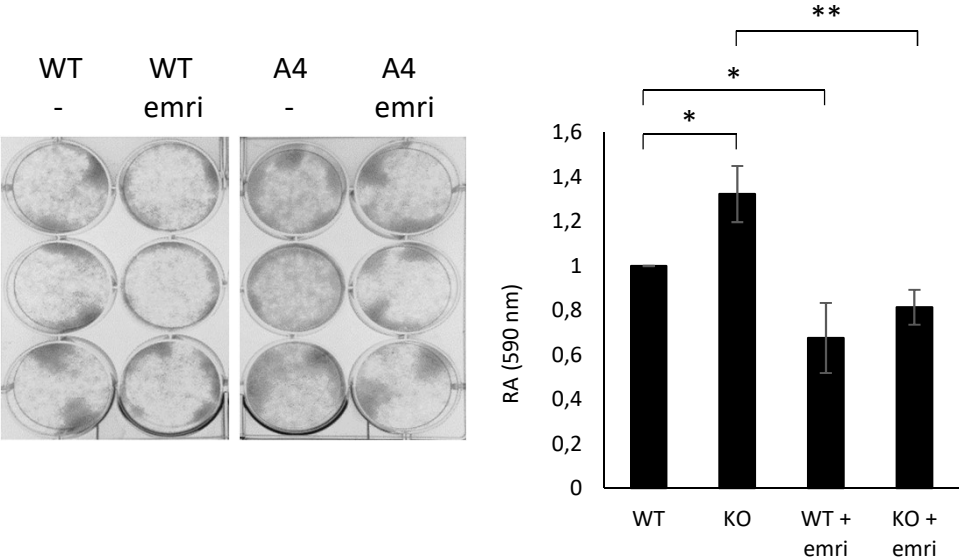

Supplement: Supplementary file 5 — Additional file 5: Figure S5. Caspases inhibition alters cell growth. WT and STAU2-KO A4 hTERT-RPE1 cells were treated with the pan-caspase inhibitor emricasan and allow to grow for 7 days. Colony growth assays were used to monitor cell proliferation. Left: representative growth of cells plated in triplicates. Right: Quantification of cell growth from three independently performed experiments. The relative growth of wild-type cells was arbitrary fixed to 1. ** p-value ≤0.01; * p-value ≤0.05. One sample t-test. [file 12860_2021_352_MOESM5_ESM.pdf]

Supplementary Figure S6

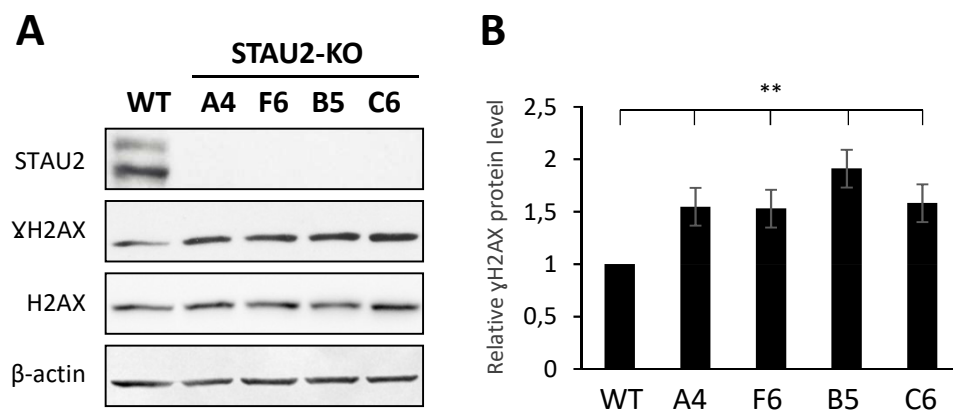

Supplement: Supplementary file 6 — Additional file 6: Figure S6. STAU2 depleted cells accumulate DNA damages. (A) Protein extracts isolated from WT and STAU2-KO hTERT-RPE1 cells were analyzed by Western Blotting for STAU2 and H2AX expression. Phosphorylated H2AX (γH2AX), a marker of DNA damages, was also revealed. The western blot is representative of three independently performed experiments. (B) Quantification of ɣH2AX protein levels in four different hTERT-RPE1 STAU2-KO cells compared to WT cells. Protein quantification represents the means and standard deviation of three independently performed experiments. The ratio of ɣH2AX on H2AX in wild type (WT) cells was arbitrary fixed to 1. ** p-value ≤0.01. One sample t-test. [file 12860_2021_352_MOESM6_ESM.pdf]
