# Supplementary material for: STAU2 protein level is controlled by caspases and the CHK1 pathway and regulates cell cycle progression in the non-transformed hTERT-RPE1 cells
Source: BMC Mol Cell Biol. 2021 Mar 4;22:16. doi: 10.1186/s12860-021-00352-y (PMC7934504; doi:10.1186/s12860-021-00352-y)

FIGURE 1A

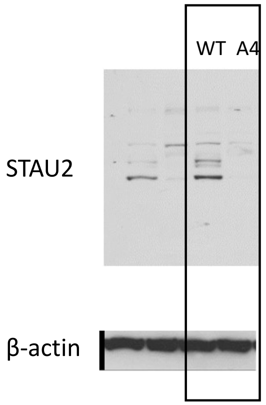

FIGURE 1D

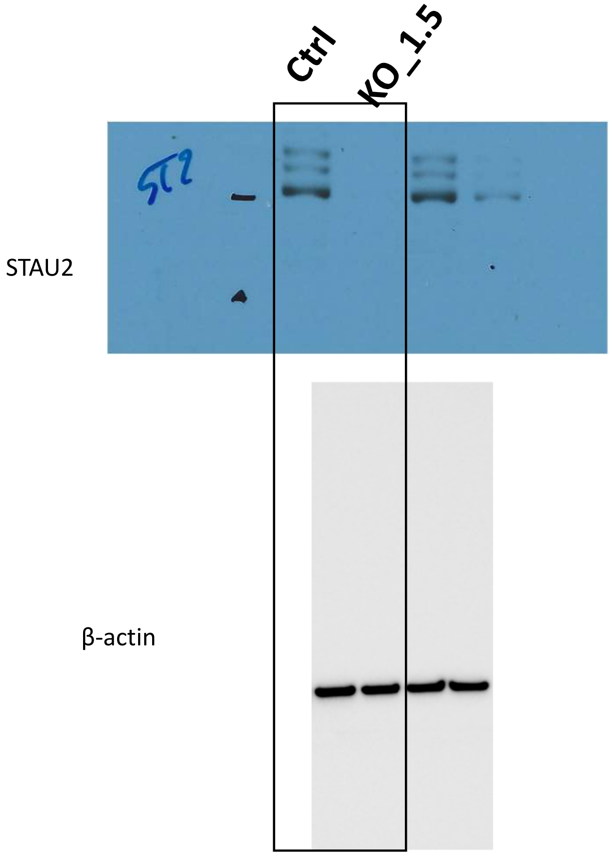

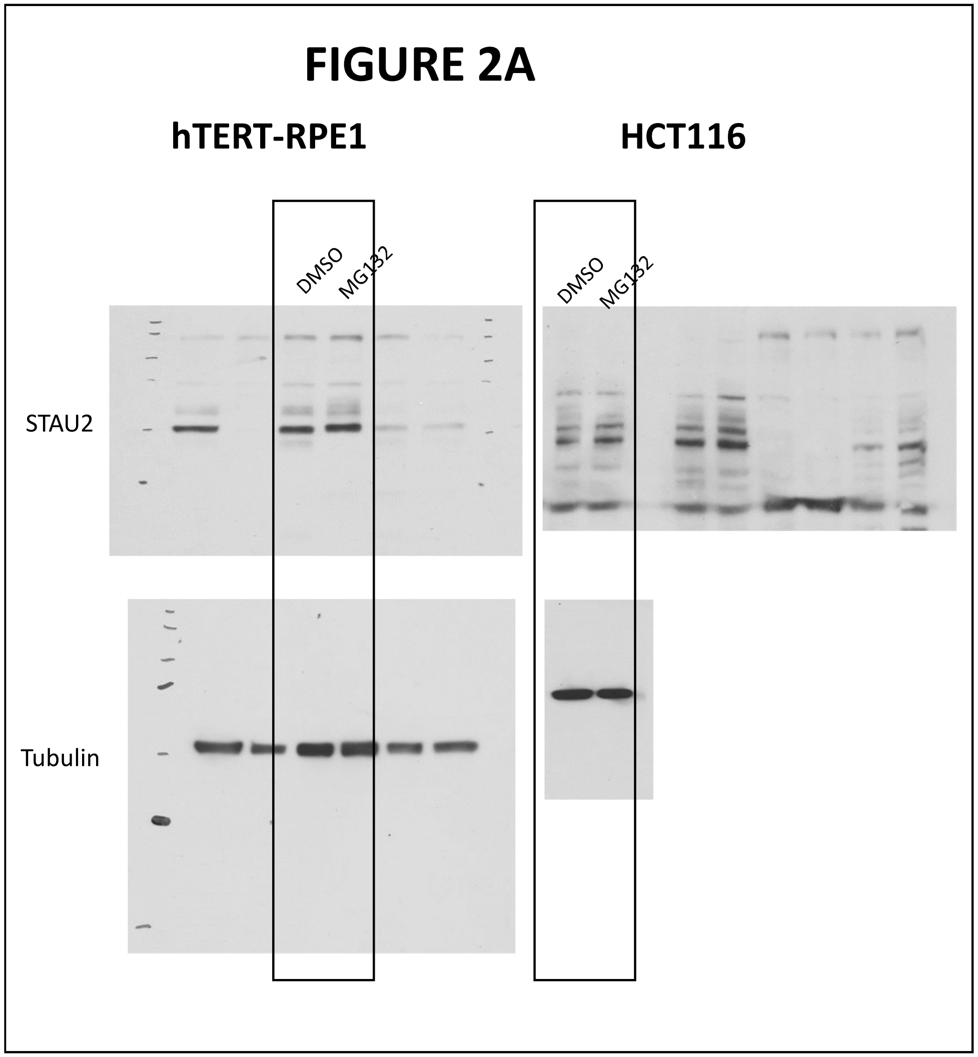
FIGURE 2A
hTERT-RPE1
HCT116
DMSO
MG132
DMSO
MG132
STAU2
Tubulin

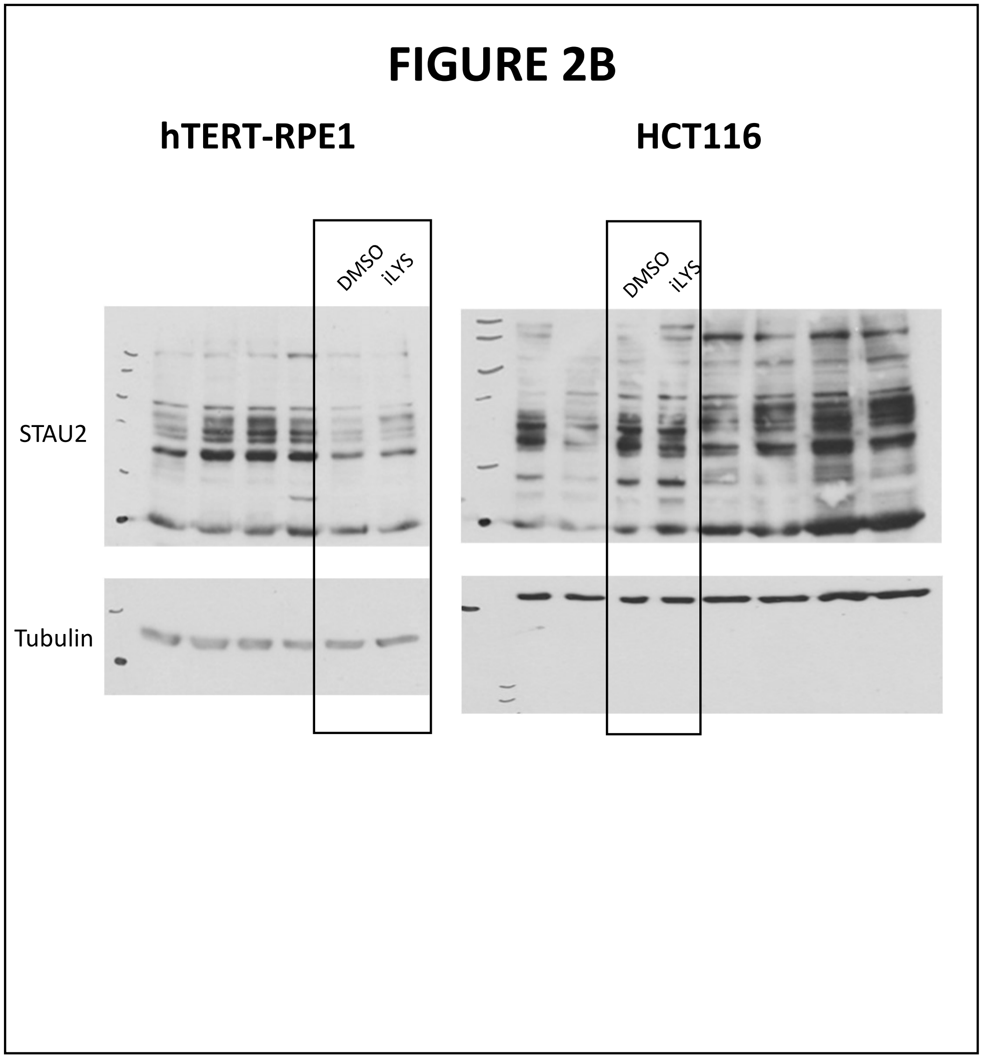
FIGURE 2B
hTERT-RPE1
HCT116
DMSO
iLYS
DMSO
iLYS
STAU2
Tubulin

FIGURE 2C

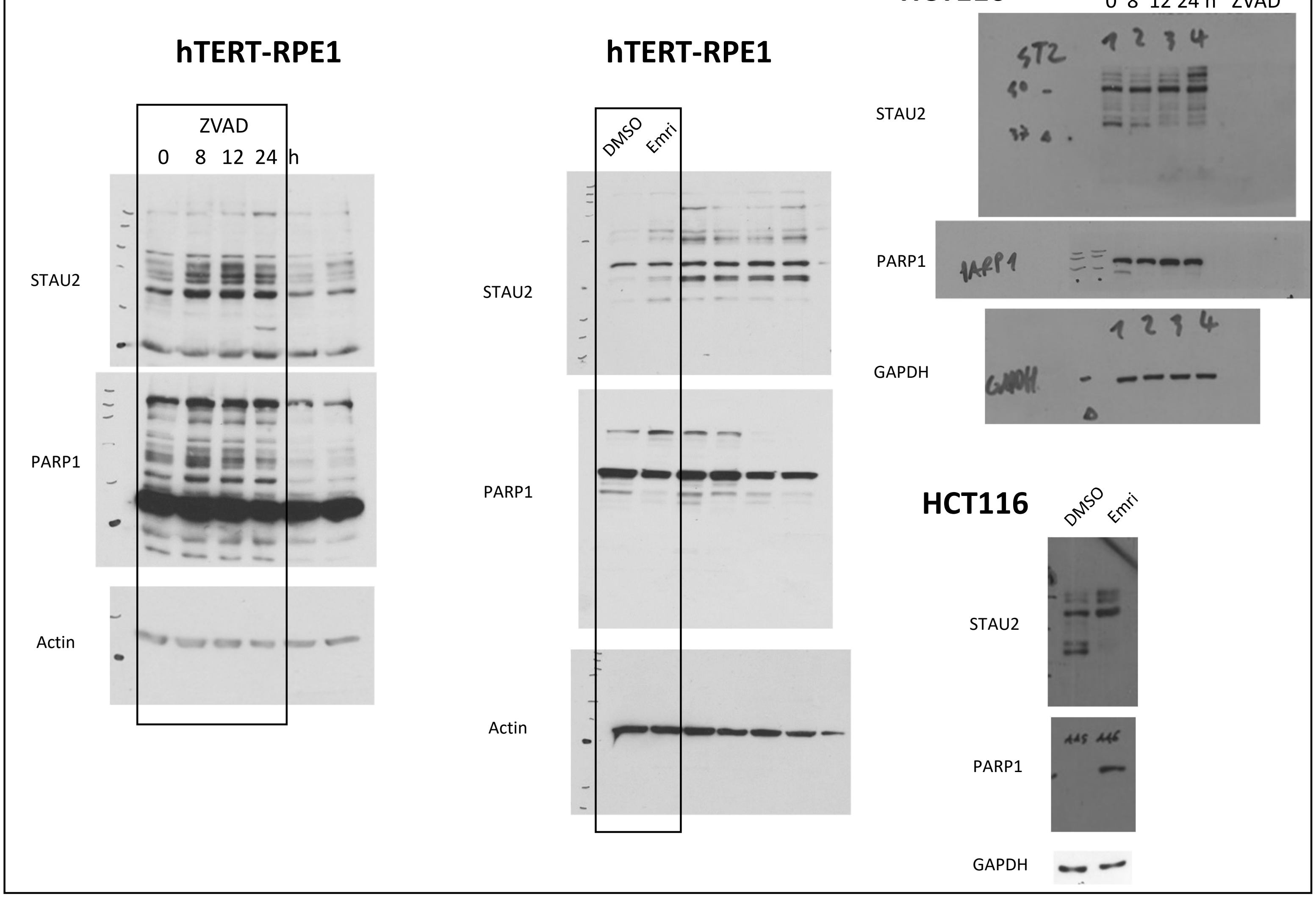

FIGURE 2D

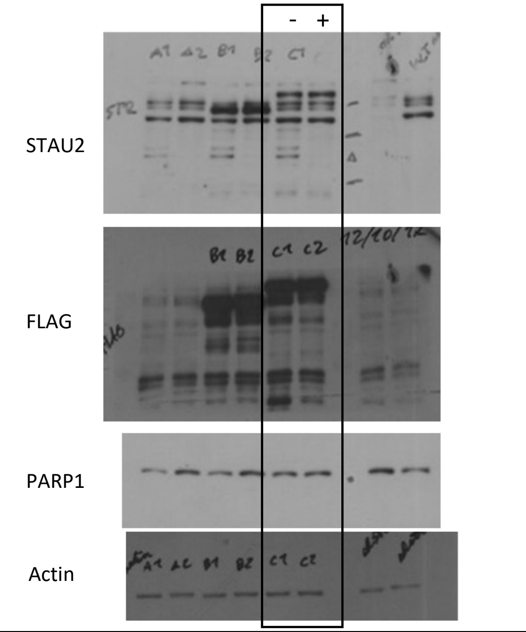

FIGURE 3B

HRP-Streptavidin

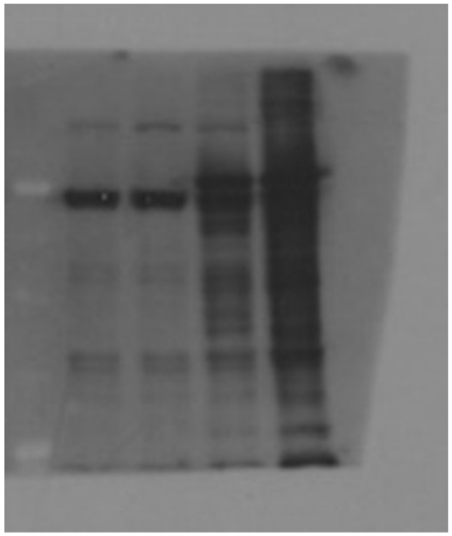

Actin

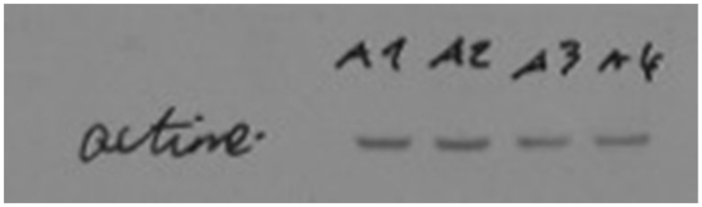

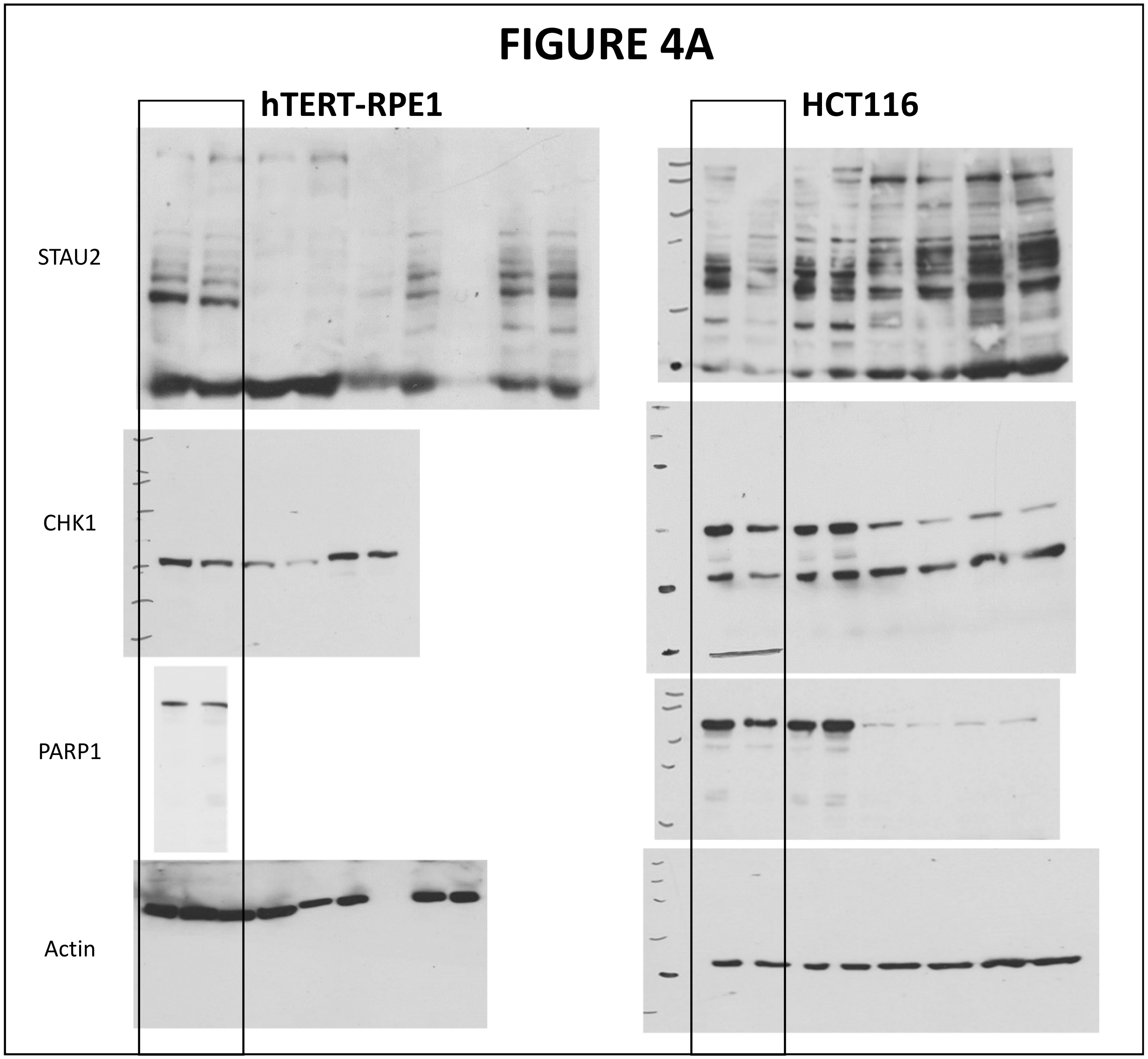
FIGURE 4A
hTERT-RPE1
HCT116
STAU2
CHK1
PARP1
Actin

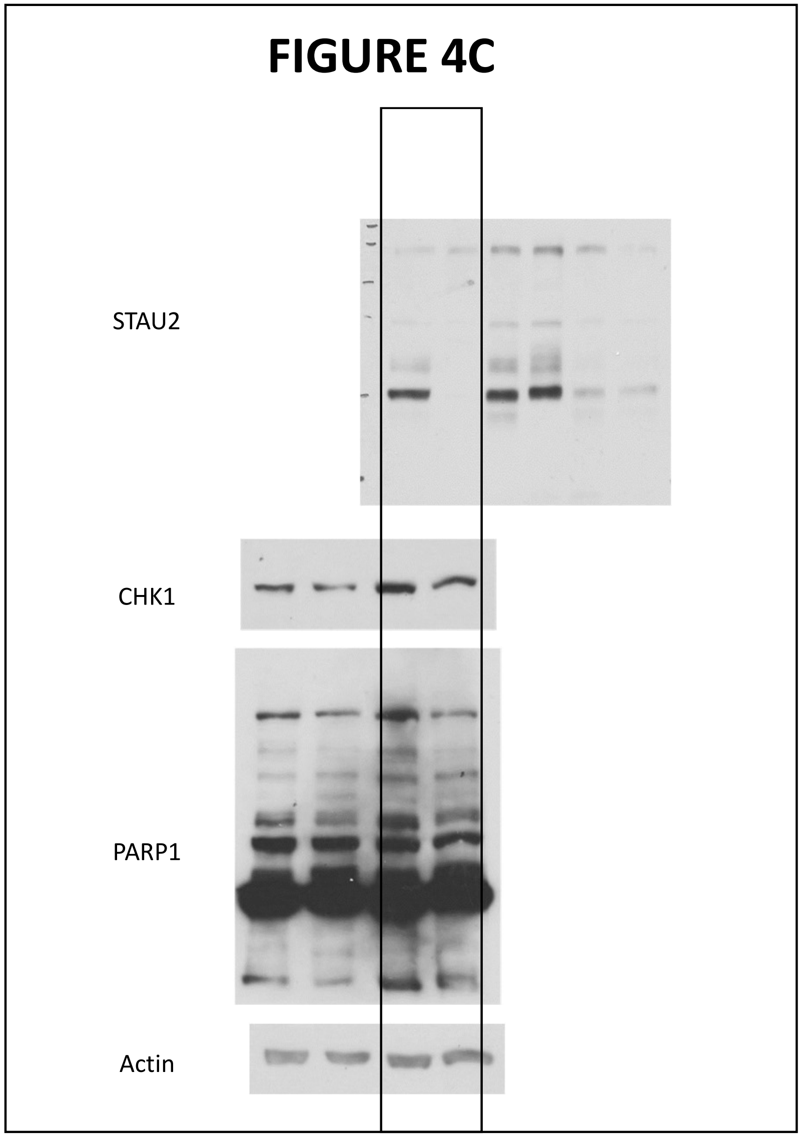
FIGURE 4C
STAU2
CHK1
PARP1
Actin

FIGURE S1 B

STAU2

Actin

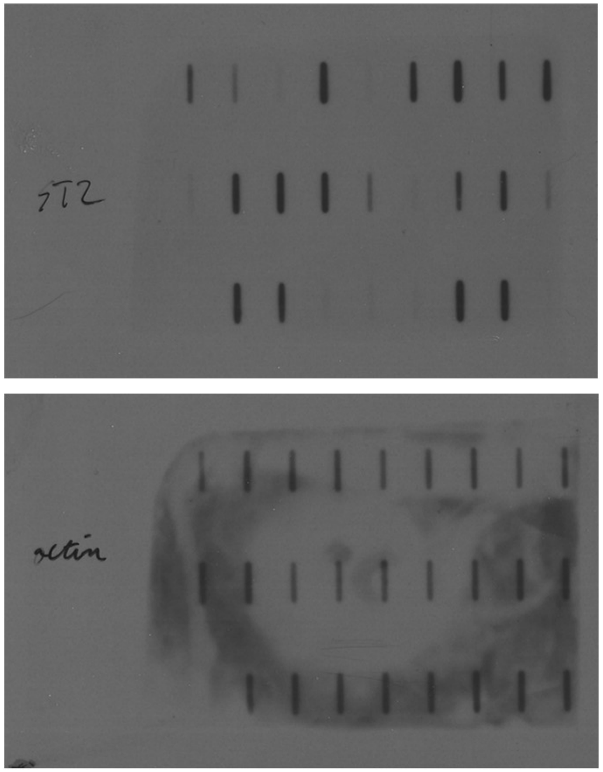

FIGURE S3 B:

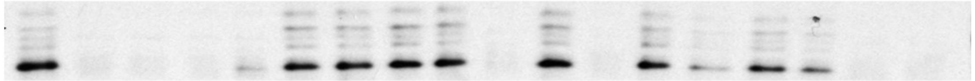

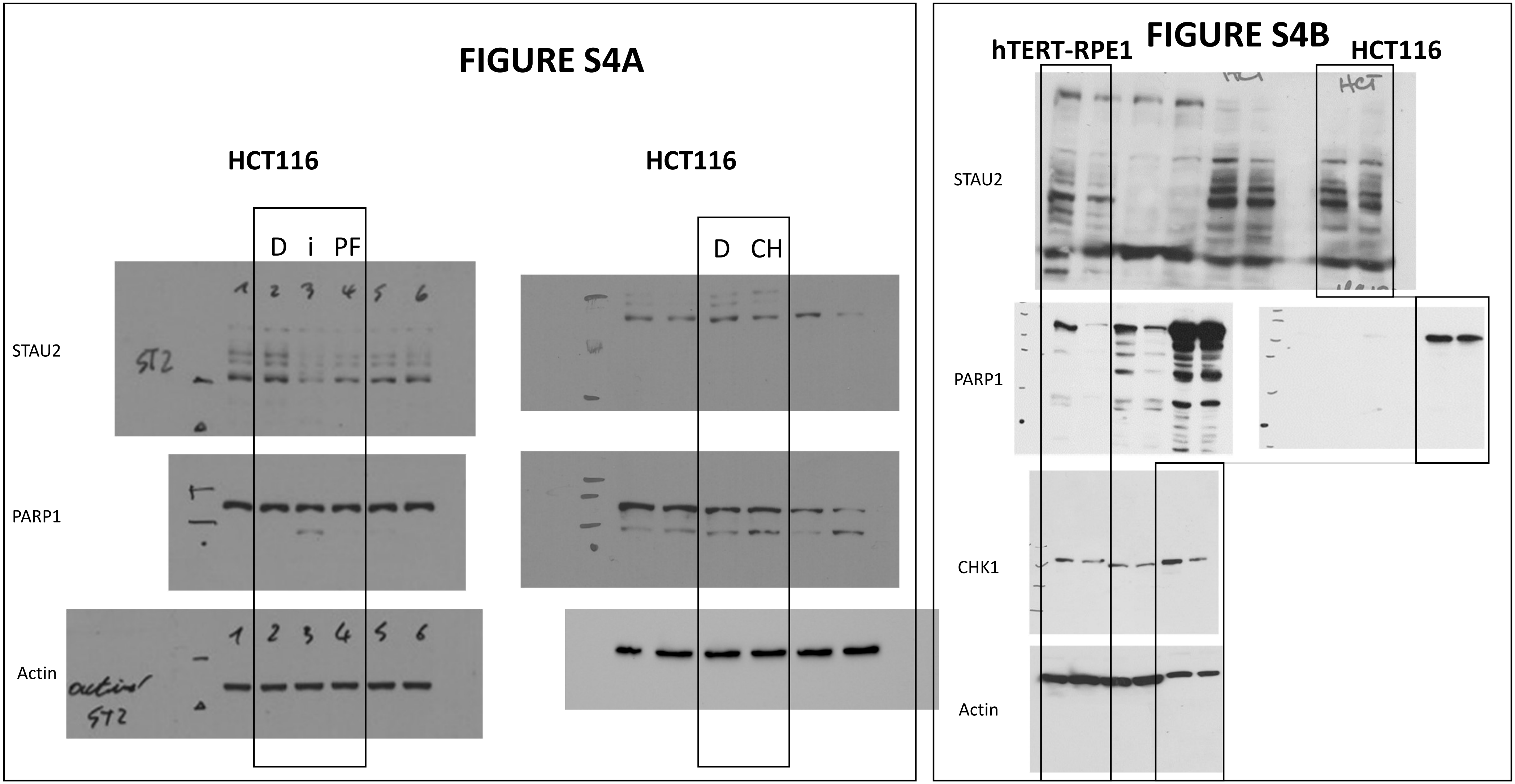
FIGURE S4A
HCT116
HCT116
D
i
PF
D
CH
STAU2
PARP1
Actin
FIGURE S4B
hTERT-RPE1
HCT116
STAU2
PARP1
CHK1
Actin

FIGURE S6 A

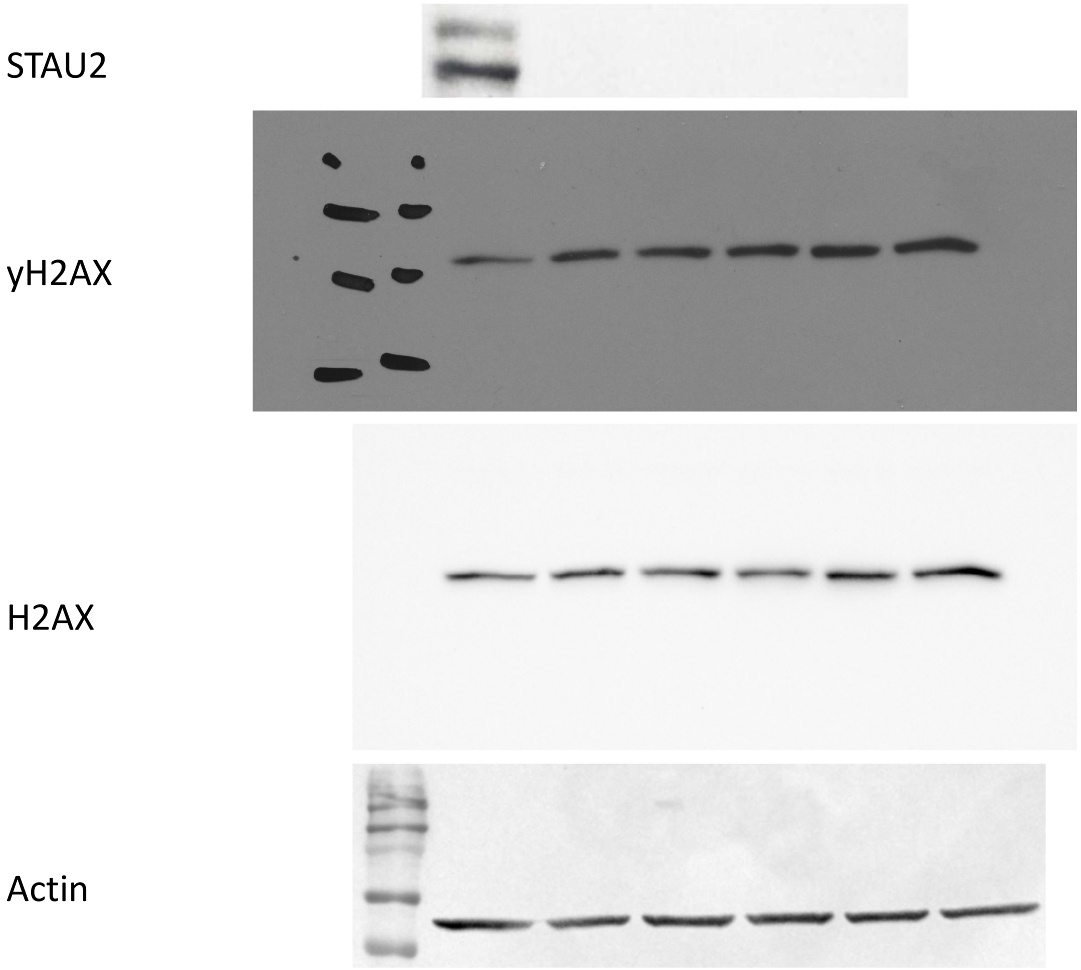

Supplement: Supplementary file 7 — Additional file 7: Figure S7. Uncropped images of immunoblots used in figures. [file 12860_2021_352_MOESM7_ESM.pdf]
