# Supplementary material for: STAU2 protein level is controlled by caspases and the CHK1 pathway and regulates cell cycle progression in the non-transformed hTERT-RPE1 cells
Source: BMC Mol Cell Biol. 2021 Mar 4;22:16. doi: 10.1186/s12860-021-00352-y (PMC7934504; doi:10.1186/s12860-021-00352-y)
Supplement: Supplementary file 11 — Additional file 11: Table S4. List of STAU2 interactors involved in DNA repair and/or replication pathways. Proteins linked to the CHK1 pathway are indicated. [file 12860_2021_352_MOESM11_ESM.pdf]

Supplementary Table S4

Proteins involved in the DNA repair and/or replication pathways

PREY	Damage Repair						Cellular Response			CHK1 PATHWAY
APEX1					BER					X
AURKB								Cell cycle		X
AQR				NER						
CAD									Others	
CCAR2									Others	
CDK1							Replication	Cell cycle		X
CDK16								Cell cycle		
CNOT1								Cell cycle	Others	
CUL4A				NER				Cell cycle	Others	X
CUL4B				NER				Cell cycle	Others	X
DDX1	DSB									
DNAJA1									Others	
FEN1	DSB	HR					Replication			X
FMR1									Others	
HUWE1					BER					
IFI16									Others	
MCM2	DSB	HR					Replication			
MCM3	DSB	HR					Replication			
MCM4	DSB	HR					Replication			
MCM5	DSB	HR					Replication			
MCM6	DSB	HR					Replication			X
MCM7	DSB	HR					Replication			X
MMS19				NER						
MSH2	DSB					MMR				X
MSH6	DSB					MMR				X
OTUB1									Others	
PARP1	DSB	HR		NER						
POLD1				NER	BER		Replication			
PRKDC	DSB		NHEJ						Others	X
RECQL	DSB	HR					Replication			X
RFC1				NER			Replication			
RFC5				NER			Replication			
RPA1	DSB	HR		NER	BER	MMR	Replication			X
RPL26									Others	
RSL1D1									Others	
RUVBL1									Others	
RUVBL2									Others	
SMCHD1	DSB									
TNKS1BP1	DSB									
TRIP13	DSB									X
XRCC5	DSB		NHEJ							X
XRCC6	DSB		NHEJ							X
YWHAG	DSB							Cell cycle		X
